# (η^6^-*p*-Cymene)(1,3-dimesityl-2,3-dihydro-1*H*-imidazol-2-yl­idene)bis­(pentafluorobenzoato-κ*O*)ruthenium(II) dichloro­methane disolvate

**DOI:** 10.1107/S1600536812049513

**Published:** 2012-12-08

**Authors:** Konstantin Dorst, Wolfgang Frey, Dongren Wang, Michael Buchmeiser

**Affiliations:** a Institut für Polymerchemie, Universität Stuttgart, Pfaffenwaldring 55, D-70569 Stuttgart, Germany; b Institut für Organische Chemie, Universität Stuttgart, Pfaffenwaldring 55, D-70569 Stuttgart, Germany

## Abstract

The title compound, [Ru(C_7_F_5_O_2_)_2_(C_10_H_14_)(C_21_H_24_N_2_)]·2CH_2_Cl_2_, is formed as an orange crystalline powder by the reaction of RuCl_2_(*p*-cymene)(IMes) and AgOCOC_6_F_5_ in anhydrous tetra­hydro­furan (IMes = 1,3-dimesityl-2,3-di­hydro-1*H*-imidazol-2-yl­idene). The asymetric unit consists of two independent [Ru(C_6_F_5_COO)_2_(η^6^-*p*-cymene)(IMes)] com­plexes and four dichloro­methane solvent mol­ecules. In each complex molecule, the Ru atom presents a pseudo-octa­hedral environment with the *p*-cymene ligand occupying three facial coordination sites, while the remaining coordination positions are occupied by the O atoms of the penta­fluoro­benzoate ligands and by the imidazolyl­idene ligand.

## Related literature

For general background to latent olefin metathesis catalysts, see: Monsaert *et al.* (2009 ▶). For ring-closing metathesis (RCM) studies of Ru^II^-*p*-cymene, see: Jafarpour *et al.* (1999 ▶). For related synthetic procedures and structures, see: Zhang *et al.* (2006 ▶); Buchmeiser *et al.* (2007 ▶). For their applications in UV-triggerable-ROMP, see: Wang *et al.* (2008 ▶, 2010 ▶).
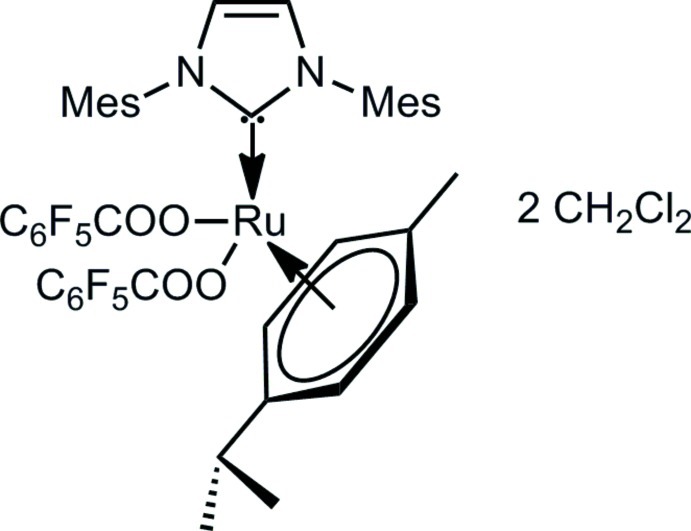


## Experimental

### 

#### Crystal data

[Ru(C_7_F_5_O_2_)_2_(C_10_H_14_)(C_21_H_24_N_2_)]·2CH_2_Cl_2_
*M* *_r_* = 1131.70
Monoclinic, 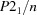

*a* = 18.0951 (11) Å
*b* = 22.4608 (14) Å
*c* = 23.4293 (14) Å
β = 104.782 (3)°
*V* = 9207.2 (10) Å^3^
*Z* = 8
Mo *K*α radiation
μ = 0.66 mm^−1^
*T* = 100 K
0.63 × 0.30 × 0.16 mm

#### Data collection

Bruker Kappa APEXII DUO diffractometer
Absorption correction: multi-scan (Blessing, 1995 ▶) *T*_min_ = 0.653, *T*_max_ = 0.746
88410 measured reflections
21150 independent reflections
16066 reflections with *I* > 2σ(*I*)
*R*_int_ = 0.037

#### Refinement

*R*[*F* ^2^ > 2σ(*F* ^2^)] = 0.059
*wR*(*F* ^2^) = 0.164
*S* = 1.09
21150 reflections
1243 parameters
H-atom parameters constrained
Δρ_max_ = 5.08 e Å^−3^
Δρ_min_ = −2.32 e Å^−3^

### 

Data collection: *APEX2* (Bruker, 2008 ▶); cell refinement: *SAINT* (Bruker, 2008 ▶); data reduction: *SAINT*; program(s) used to solve structure: *SHELXS97* (Sheldrick, 2008 ▶); program(s) used to refine structure: *SHELXL97* (Sheldrick, 2008 ▶); molecular graphics: *XP* in *SHELXTL-Plus* (Sheldrick, 2008 ▶); software used to prepare material for publication: *publCIF* (Westrip, 2010 ▶).

## Supplementary Material

Crystal structure: contains datablock(s) I, global. DOI: 10.1107/S1600536812049513/im2412sup1.cif

Structure factors: contains datablock(s) I. DOI: 10.1107/S1600536812049513/im2412Isup2.hkl

Additional supplementary materials:  crystallographic information; 3D view; checkCIF report

## Figures and Tables

**Fig. 1.**
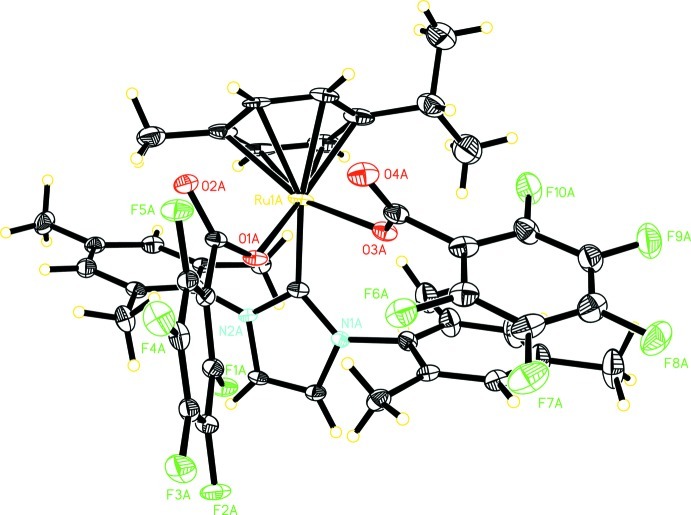
Molecular structure of the title molecule with displacement ellipsoids drawn at the 50% probability level. H atoms have been omitted for clarity.

**Fig. 2.**
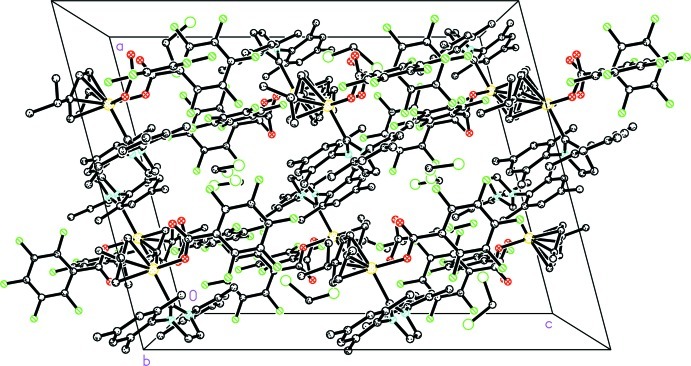
A view of the unit-cell contents of the title compound.

## supplementary crystallographic information

### Comment

In the last years, the design and synthesis of latent olefin metathesis precatalysts has received considerable attention. Due to their latency, they exhibit no catalytic activity at low (usually ambient) temperature in the presence of monomer or substrate, but can be triggered quantitatively to a highly active form. This activation can be caused by a variety of different stimuli, *e.g.*, light activation to initiate metathesis reactions. Such precatalysts are of particular interest in technical applications of ROMP, since they allow for being mixed with the monomers, its storage over a longer period of time even at elevated temperatures without concomitant polymerization (Buchmeiser *et al.* 2007, Monsaert *et al.* 2009). The title compound (Figure 1), an intermediate product for the synthesis of a photo-latent metathesis catalyst, was obtained by the reaction of RuCl_2_(*p*-cymene)(IMes) and AgOCOC_6_F_5_ in anhydrous THF (IMes = 1,3-dimesityl-2,3-dihydro-1*H*-imidazol-2-ylidene). It crystallizes in the space group P2_1_/n (*Z*=8) with accompanying CH_2_Cl_2_ solvate molecules. Selected geometric parameters are given in table 1. The structure exhibits a typical three legged piano stool structure with RuII coordinated by one carbon atom of the N-heterocyclic carbene ligand and two oxygen atoms of the pentafluorobenzoate ligands. The coordination geometry around Ru is that of a distorted octahedron in which three sites are occupied by the η^6^-*p*-cymene ligand, two by the pentafluorobenzoate ligands and one by the N-heterocyclic carbene. The structure is closely similar to that of recently reported compound Ru(CF_3_COO)(*p*-cymene)(IMes) (Zhang *et al.* 2006).

### Experimental

A solution of silver pentafluorobenzoate (356.9 mg, 1.12 mmol) in 10 ml of anhydrous THF was added to a solution of RuCl_2_(IMes)(*p*-cymene) (328.5 mg, 0.54 mmol) in 10 ml of anhydrous THF under stirring. After 4 h the mixture was filtered through a short bed of celite and THF was removed *in vacuo*. Dichloromethane was added to dissolve the residues and the solution was filtered through glass-fiber paper and concentrated *in vacu*o. Diethyl ether was layered over the red, saturated CH_2_Cl_2_ solution. Red crystals suitable for X-ray analysis formed at -36°C. Yield: 310 mg (60%).

### Refinement

H atoms were located from difference fourier maps, but were refined with fixed individual displacement parameters [U_iso_(H) = 1.2 *U*_eq_(C) or U(H) = 1.5 *U*_eq_(*C*-methyl-)] using a riding model with C—H ranging from 0.95 to 1.0 Å. In addition, the methyl groups were allowed to rotate but not to tip.

### Crystal data

**Table:**

[Ru(C_7_F_5_O_2_)_2_(C_10_H_14_)(C_21_H_24_N_2_)]·2CH_2_Cl_2_	*F*(000) = 4576
*M**_r_* = 1131.70	*D*_x_ = 1.633 Mg m^−^^3^
Monoclinic, *P*2_1_/*n*	Mo *K*α radiation, λ = 0.71073 Å
Hall symbol: -P 2yn	Cell parameters from 16066 reflections
*a* = 18.0951 (11) Å	θ = 1.8–27.5°
*b* = 22.4608 (14) Å	µ = 0.66 mm^−^^1^
*c* = 23.4293 (14) Å	*T* = 100 K
β = 104.782 (3)°	Plates, orange
*V* = 9207.2 (10) Å^3^	0.63 × 0.30 × 0.16 mm
*Z* = 8	

### Data collection

**Table:**

Bruker Kappa APEXII DUO diffractometer	21150 independent reflections
Radiation source: fine-focus sealed tube	16066 reflections with *I* > 2σ(*I*)
Triumph monochromator	*R*_int_ = 0.037
ω + Phi Scans scans	θ_max_ = 27.5°, θ_min_ = 1.8°
Absorption correction: multi-scan (Blessing, 1995)	*h* = −23→12
*T*_min_ = 0.653, *T*_max_ = 0.746	*k* = −26→29
88410 measured reflections	*l* = −30→30

### Refinement

**Table:**

Refinement on *F*^2^	Primary atom site location: structure-invariant direct methods
Least-squares matrix: full	Secondary atom site location: difference Fourier map
*R*[*F*^2^ > 2σ(*F*^2^)] = 0.059	Hydrogen site location: inferred from neighbouring sites
*wR*(*F*^2^) = 0.164	H-atom parameters constrained
*S* = 1.09	*w* = 1/[σ^2^(*F*_o_^2^) + (0.0866*P*)^2^ + 19.1814*P*] where *P* = (*F*_o_^2^ + 2*F*_c_^2^)/3
21150 reflections	(Δ/σ)_max_ = 0.001
1243 parameters	Δρ_max_ = 5.08 e Å^−^^3^
0 restraints	Δρ_min_ = −2.32 e Å^−^^3^

### Special details

**Table:**

Geometry. All e.s.d.'s (except the e.s.d. in the dihedral angle between two l.s. planes) are estimated using the full covariance matrix. The cell e.s.d.'s are taken into account individually in the estimation of e.s.d.'s in distances, angles and torsion angles; correlations between e.s.d.'s in cell parameters are only used when they are defined by crystal symmetry. An approximate (isotropic) treatment of cell e.s.d.'s is used for estimating e.s.d.'s involving l.s. planes.
Refinement. Refinement of *F*^2^ against ALL reflections. The weighted *R*-factor *wR* and goodness of fit *S* are based on *F*^2^, conventional *R*-factors *R* are based on *F*, with *F* set to zero for negative *F*^2^. The threshold expression of *F*^2^ > σ(*F*^2^) is used only for calculating *R*-factors (gt) *etc*. and is not relevant to the choice of reflections for refinement. *R*-factors based on *F*^2^ are statistically about twice as large as those based on *F*, and *R*– factors based on ALL data will be even larger.

### Fractional atomic coordinates and isotropic or equivalent isotropic displacement parameters (Å^2^)

**Table:**

	*x*	*y*	*z*	*U*_iso_*/*U*_eq_	
Ru1A	0.211201 (14)	0.733381 (14)	0.034965 (12)	0.01547 (8)	
F1A	0.19263 (11)	0.80651 (10)	0.20855 (9)	0.0201 (4)	
F2A	0.22409 (13)	0.80990 (11)	0.32495 (10)	0.0267 (5)	
F3A	0.37020 (14)	0.82051 (11)	0.39036 (9)	0.0271 (5)	
F4A	0.48626 (12)	0.82406 (11)	0.33637 (10)	0.0262 (5)	
F5A	0.45810 (11)	0.81535 (11)	0.21975 (10)	0.0244 (5)	
F6A	0.33768 (13)	0.64401 (11)	0.24677 (11)	0.0310 (5)	
F7A	0.33828 (15)	0.54675 (13)	0.31401 (11)	0.0391 (6)	
F8A	0.29924 (15)	0.43914 (12)	0.26195 (12)	0.0383 (6)	
F9A	0.26109 (17)	0.42970 (12)	0.14284 (12)	0.0419 (7)	
F10A	0.25607 (16)	0.52702 (11)	0.07608 (11)	0.0351 (6)	
O1A	0.24672 (13)	0.77272 (11)	0.11899 (10)	0.0151 (5)	
O2A	0.35391 (14)	0.82194 (12)	0.11620 (12)	0.0211 (6)	
O3A	0.22999 (13)	0.65765 (12)	0.09000 (11)	0.0184 (5)	
O4A	0.35781 (14)	0.66912 (13)	0.12402 (12)	0.0254 (6)	
N1A	0.07045 (15)	0.70974 (13)	0.08909 (12)	0.0127 (6)	
N2A	0.06483 (15)	0.79737 (13)	0.05245 (12)	0.0121 (5)	
C1A	0.10574 (18)	0.74578 (16)	0.05680 (15)	0.0137 (7)	
C2A	0.01060 (19)	0.73846 (16)	0.10435 (16)	0.0161 (7)	
H2A	−0.0216	0.7223	0.1268	0.019*	
C3A	0.00678 (18)	0.79328 (16)	0.08153 (15)	0.0150 (7)	
H3A	−0.0287	0.8236	0.0845	0.018*	
C4A	0.3009 (2)	0.70314 (18)	−0.00775 (16)	0.0205 (8)	
H4A	0.3444	0.6785	0.0065	0.025*	
C5A	0.3088 (2)	0.76397 (18)	0.00058 (16)	0.0202 (8)	
H5A	0.3568	0.7802	0.0208	0.024*	
C6A	0.2451 (2)	0.80253 (19)	−0.02105 (17)	0.0260 (9)	
C7A	0.1747 (2)	0.7749 (2)	−0.05192 (16)	0.0316 (11)	
H7A	0.1318	0.7995	−0.0681	0.038*	
C8A	0.1666 (2)	0.7130 (2)	−0.05920 (17)	0.0292 (9)	
H8A	0.1185	0.6964	−0.0790	0.035*	
C9A	0.2294 (2)	0.6757 (2)	−0.03724 (17)	0.0260 (9)	
C10A	0.2547 (3)	0.8678 (2)	−0.0141 (2)	0.0348 (11)	
H10A	0.2791	0.8772	0.0272	0.052*	
H10B	0.2046	0.8871	−0.0259	0.052*	
H10C	0.2869	0.8822	−0.0391	0.052*	
C11A	0.2284 (3)	0.6079 (2)	−0.0443 (2)	0.0325 (10)	
H11A	0.2649	0.5912	−0.0084	0.039*	
C12A	0.2608 (3)	0.5937 (2)	−0.0968 (2)	0.0459 (13)	
H12A	0.2591	0.5506	−0.1036	0.069*	
H12B	0.3138	0.6075	−0.0886	0.069*	
H12C	0.2302	0.6138	−0.1321	0.069*	
C13A	0.1524 (3)	0.5774 (2)	−0.0500 (3)	0.0459 (13)	
H13A	0.1344	0.5855	−0.0148	0.069*	
H13B	0.1585	0.5344	−0.0541	0.069*	
H13C	0.1152	0.5927	−0.0850	0.069*	
C14A	0.07930 (19)	0.64673 (16)	0.10204 (15)	0.0164 (7)	
C15A	0.0405 (2)	0.60653 (19)	0.05989 (18)	0.0281 (9)	
C16A	0.0422 (3)	0.5467 (2)	0.0756 (2)	0.0350 (10)	
H16A	0.0167	0.5185	0.0472	0.042*	
C17A	0.0797 (3)	0.52701 (19)	0.13108 (19)	0.0305 (9)	
C18A	0.11557 (19)	0.56887 (17)	0.17216 (16)	0.0192 (7)	
H18A	0.1417	0.5558	0.2106	0.023*	
C19A	0.11490 (18)	0.62901 (16)	0.15943 (15)	0.0158 (7)	
C20A	−0.0048 (3)	0.6271 (2)	0.00029 (19)	0.0339 (10)	
H20A	−0.0465	0.6531	0.0049	0.051*	
H20B	−0.0262	0.5926	−0.0238	0.051*	
H20C	0.0286	0.6493	−0.0192	0.051*	
C21A	0.0804 (3)	0.4620 (2)	0.1466 (2)	0.0429 (12)	
H21A	0.0306	0.4444	0.1279	0.064*	
H21B	0.0909	0.4576	0.1895	0.064*	
H21C	0.1203	0.4417	0.1324	0.064*	
C22A	0.1509 (2)	0.67352 (18)	0.20563 (16)	0.0211 (8)	
H22A	0.1774	0.6527	0.2418	0.032*	
H22B	0.1112	0.6994	0.2139	0.032*	
H22C	0.1876	0.6977	0.1915	0.032*	
C23A	0.06434 (18)	0.84920 (15)	0.01554 (15)	0.0133 (6)	
C24A	0.10107 (18)	0.90065 (16)	0.04012 (15)	0.0157 (7)	
C25A	0.0915 (2)	0.95158 (17)	0.00481 (17)	0.0198 (7)	
H25A	0.1165	0.9873	0.0208	0.024*	
C26A	0.0469 (2)	0.95187 (17)	−0.05264 (17)	0.0191 (7)	
C27A	0.00898 (19)	0.89935 (17)	−0.07535 (16)	0.0186 (7)	
H27A	−0.0224	0.8990	−0.1146	0.022*	
C28A	0.01624 (18)	0.84773 (16)	−0.04166 (15)	0.0147 (7)	
C29A	−0.02639 (19)	0.79270 (17)	−0.06700 (16)	0.0180 (7)	
H29A	−0.0622	0.8022	−0.1049	0.027*	
H29B	−0.0548	0.7774	−0.0396	0.027*	
H29C	0.0099	0.7624	−0.0730	0.027*	
C30A	0.0376 (2)	1.00646 (18)	−0.09032 (19)	0.0269 (9)	
H30A	0.0600	1.0407	−0.0661	0.040*	
H30B	−0.0168	1.0138	−0.1078	0.040*	
H30C	0.0637	1.0007	−0.1218	0.040*	
C31A	0.1479 (2)	0.90321 (17)	0.10271 (17)	0.0223 (8)	
H31A	0.1144	0.8987	0.1294	0.033*	
H31B	0.1743	0.9416	0.1100	0.033*	
H31C	0.1857	0.8710	0.1098	0.033*	
C32A	0.30839 (18)	0.80074 (16)	0.14130 (15)	0.0143 (7)	
C33A	0.32401 (19)	0.80765 (15)	0.20791 (15)	0.0141 (7)	
C34A	0.26638 (19)	0.80762 (16)	0.23782 (16)	0.0159 (7)	
C35A	0.2813 (2)	0.81073 (17)	0.29843 (16)	0.0184 (7)	
C36A	0.3557 (2)	0.81628 (16)	0.33167 (15)	0.0182 (7)	
C37A	0.41394 (19)	0.81774 (16)	0.30390 (16)	0.0177 (7)	
C38A	0.39800 (19)	0.81357 (16)	0.24320 (16)	0.0161 (7)	
C39A	0.2966 (2)	0.64470 (17)	0.12117 (16)	0.0197 (7)	
C40A	0.29610 (19)	0.58889 (19)	0.15947 (17)	0.0217 (8)	
C41A	0.3175 (2)	0.59184 (19)	0.22004 (17)	0.0234 (8)	
C42A	0.3184 (2)	0.5420 (2)	0.25526 (17)	0.0265 (9)	
C43A	0.2987 (2)	0.4875 (2)	0.22897 (19)	0.0293 (9)	
C44A	0.2792 (2)	0.48252 (19)	0.16859 (19)	0.0284 (9)	
C45A	0.2765 (2)	0.53375 (18)	0.13436 (18)	0.0253 (8)	
Ru1B	0.216163 (14)	0.751183 (12)	0.535681 (11)	0.01067 (8)	
F1B	0.33746 (13)	0.65939 (11)	0.74261 (10)	0.0269 (5)	
F2B	0.34588 (14)	0.56074 (13)	0.80945 (10)	0.0362 (6)	
F3B	0.31731 (15)	0.45214 (12)	0.75748 (12)	0.0394 (7)	
F4B	0.28407 (16)	0.44301 (11)	0.63900 (13)	0.0404 (6)	
F5B	0.26975 (15)	0.54057 (11)	0.57176 (11)	0.0325 (6)	
F6B	0.19288 (11)	0.81864 (10)	0.71256 (9)	0.0186 (4)	
F7B	0.22163 (12)	0.81990 (10)	0.82943 (10)	0.0235 (5)	
F8B	0.36714 (13)	0.81805 (11)	0.89705 (9)	0.0254 (5)	
F9B	0.48511 (12)	0.81307 (12)	0.84560 (10)	0.0287 (5)	
F10B	0.45904 (12)	0.80537 (12)	0.72857 (10)	0.0276 (5)	
O1B	0.23489 (13)	0.67194 (11)	0.58540 (11)	0.0152 (5)	
O2B	0.36216 (13)	0.68584 (12)	0.62238 (12)	0.0214 (6)	
O3B	0.24842 (12)	0.78394 (11)	0.62251 (10)	0.0144 (5)	
O4B	0.35836 (14)	0.83282 (12)	0.62617 (11)	0.0188 (5)	
N1B	0.06452 (15)	0.80710 (13)	0.55291 (12)	0.0128 (6)	
N2B	0.07508 (15)	0.71824 (13)	0.58685 (12)	0.0118 (5)	
C1B	0.10899 (18)	0.75737 (15)	0.55659 (15)	0.0127 (6)	
C2B	0.00643 (18)	0.79907 (17)	0.58090 (15)	0.0163 (7)	
H2B	−0.0307	0.8278	0.5845	0.020*	
C3B	0.01232 (18)	0.74379 (17)	0.60178 (16)	0.0165 (7)	
H3B	−0.0199	0.7253	0.6228	0.020*	
C4B	0.1875 (2)	0.80780 (17)	0.45545 (15)	0.0186 (7)	
H4B	0.1483	0.8366	0.4426	0.022*	
C5B	0.1723 (2)	0.74728 (18)	0.44022 (16)	0.0192 (8)	
H5B	0.1223	0.7359	0.4188	0.023*	
C6B	0.22997 (19)	0.70319 (17)	0.45620 (15)	0.0178 (7)	
C7B	0.30300 (18)	0.72224 (17)	0.49023 (15)	0.0157 (7)	
H7B	0.3430	0.6939	0.5018	0.019*	
C8B	0.31720 (18)	0.78133 (16)	0.50697 (15)	0.0156 (7)	
H8B	0.3661	0.7923	0.5307	0.019*	
C9B	0.2596 (2)	0.82594 (16)	0.48914 (15)	0.0168 (7)	
C10B	0.2772 (2)	0.89004 (17)	0.50338 (18)	0.0244 (8)	
H10D	0.3059	0.9063	0.4767	0.037*	
H10E	0.3077	0.8937	0.5443	0.037*	
H10F	0.2293	0.9123	0.4985	0.037*	
C11B	0.2146 (2)	0.63985 (19)	0.43595 (18)	0.0266 (9)	
H11B	0.1745	0.6229	0.4538	0.032*	
C12B	0.2846 (3)	0.6005 (2)	0.4520 (2)	0.0389 (11)	
H12D	0.3248	0.6175	0.4358	0.058*	
H12E	0.2715	0.5606	0.4354	0.058*	
H12F	0.3028	0.5977	0.4950	0.058*	
C13B	0.1843 (4)	0.6393 (3)	0.3682 (2)	0.0581 (17)	
H13D	0.1350	0.6599	0.3569	0.087*	
H13E	0.1777	0.5980	0.3542	0.087*	
H13F	0.2209	0.6595	0.3504	0.087*	
C14B	0.06306 (18)	0.85923 (16)	0.51688 (15)	0.0146 (7)	
C15B	0.01518 (19)	0.85851 (17)	0.45963 (16)	0.0171 (7)	
C16B	0.0105 (2)	0.90990 (18)	0.42574 (17)	0.0211 (8)	
H16B	−0.0213	0.9100	0.3866	0.025*	
C17B	0.0512 (2)	0.96122 (18)	0.44769 (18)	0.0239 (8)	
C18B	0.0951 (2)	0.96080 (17)	0.50537 (18)	0.0226 (8)	
H18B	0.1214	0.9961	0.5212	0.027*	
C19B	0.10206 (19)	0.91061 (16)	0.54123 (16)	0.0171 (7)	
C20B	−0.02853 (19)	0.80376 (17)	0.43447 (16)	0.0195 (7)	
H20D	−0.0647	0.8136	0.3968	0.029*	
H20E	−0.0566	0.7887	0.4622	0.029*	
H20F	0.0071	0.7732	0.4280	0.029*	
C21B	0.0454 (3)	1.0153 (2)	0.4091 (2)	0.0347 (10)	
H21D	0.0719	1.0487	0.4325	0.052*	
H21E	−0.0086	1.0255	0.3930	0.052*	
H21F	0.0689	1.0069	0.3767	0.052*	
C22B	0.1496 (2)	0.91242 (17)	0.60376 (17)	0.0234 (8)	
H22D	0.1179	0.9017	0.6304	0.035*	
H22E	0.1701	0.9526	0.6131	0.035*	
H22F	0.1919	0.8841	0.6086	0.035*	
C23B	0.08804 (18)	0.65534 (15)	0.59738 (15)	0.0135 (6)	
C24B	0.06337 (19)	0.61648 (17)	0.55047 (16)	0.0182 (7)	
C25B	0.0712 (2)	0.55586 (18)	0.56177 (17)	0.0224 (8)	
H25B	0.0559	0.5287	0.5299	0.027*	
C26B	0.1006 (2)	0.53389 (17)	0.61800 (18)	0.0220 (8)	
C27B	0.12249 (19)	0.57448 (16)	0.66432 (16)	0.0173 (7)	
H27B	0.1411	0.5599	0.7034	0.021*	
C28B	0.11782 (18)	0.63565 (16)	0.65504 (15)	0.0155 (7)	
C29B	0.0265 (2)	0.6387 (2)	0.48910 (17)	0.0274 (9)	
H29D	−0.0194	0.6616	0.4896	0.041*	
H29E	0.0125	0.6048	0.4623	0.041*	
H29F	0.0626	0.6643	0.4756	0.041*	
C30B	0.1063 (3)	0.46760 (19)	0.6284 (2)	0.0322 (10)	
H30D	0.0556	0.4516	0.6274	0.048*	
H30E	0.1412	0.4596	0.6671	0.048*	
H30F	0.1259	0.4486	0.5976	0.048*	
C31B	0.1457 (2)	0.67853 (17)	0.70486 (16)	0.0179 (7)	
H31D	0.1704	0.6565	0.7408	0.027*	
H31E	0.1023	0.7012	0.7115	0.027*	
H31F	0.1826	0.7060	0.6949	0.027*	
C32B	0.30106 (19)	0.65946 (16)	0.61778 (15)	0.0162 (7)	
C33B	0.30264 (18)	0.60368 (17)	0.65504 (16)	0.0179 (7)	
C34B	0.32219 (19)	0.60660 (18)	0.71560 (17)	0.0200 (8)	
C35B	0.3272 (2)	0.5564 (2)	0.75062 (17)	0.0256 (9)	
C36B	0.3132 (2)	0.50171 (19)	0.72432 (19)	0.0272 (9)	
C37B	0.2952 (2)	0.49661 (19)	0.66437 (19)	0.0276 (9)	
C38B	0.2888 (2)	0.54743 (18)	0.63013 (17)	0.0222 (8)	
C39B	0.31051 (18)	0.80947 (16)	0.64793 (15)	0.0142 (7)	
C40B	0.32475 (18)	0.81116 (15)	0.71470 (15)	0.0135 (7)	
C41B	0.26600 (18)	0.81513 (15)	0.74324 (15)	0.0141 (7)	
C42B	0.27953 (19)	0.81664 (16)	0.80398 (16)	0.0159 (7)	
C43B	0.3537 (2)	0.81571 (16)	0.83817 (15)	0.0168 (7)	
C44B	0.41293 (19)	0.81273 (17)	0.81215 (16)	0.0185 (7)	
C45B	0.39847 (19)	0.80971 (16)	0.75135 (16)	0.0170 (7)	
C1X	0.0182 (2)	0.85437 (19)	0.73301 (18)	0.0267 (9)	
H1X1	−0.0287	0.8434	0.7025	0.032*	
H1X2	0.0621	0.8509	0.7153	0.032*	
Cl1	0.03067 (7)	0.80522 (5)	0.79314 (5)	0.0377 (3)	
Cl2	0.01052 (6)	0.92889 (5)	0.75558 (6)	0.0388 (3)	
C2X	0.0223 (2)	0.8451 (2)	0.23282 (18)	0.0266 (9)	
H2X1	−0.0255	0.8301	0.2059	0.032*	
H2X2	0.0655	0.8341	0.2162	0.032*	
Cl3	0.03471 (7)	0.81259 (7)	0.30215 (6)	0.0481 (3)	
Cl4	0.01756 (7)	0.92300 (6)	0.23876 (9)	0.0673 (5)	
C3X	0.0756 (3)	0.4518 (2)	0.8115 (2)	0.0457 (13)	
H3X1	0.0580	0.4836	0.8343	0.055*	
H3X2	0.0767	0.4139	0.8332	0.055*	
Cl5	0.16713 (7)	0.46850 (6)	0.80490 (6)	0.0465 (3)	
Cl6	0.01067 (7)	0.44556 (8)	0.74007 (6)	0.0584 (4)	
C4X	0.0896 (3)	0.4513 (2)	0.3654 (2)	0.0365 (11)	
H4X1	0.0378	0.4634	0.3428	0.044*	
H4X2	0.0881	0.4086	0.3754	0.044*	
Cl7	0.15538 (6)	0.46240 (5)	0.32162 (5)	0.0386 (3)	
Cl8	0.11683 (9)	0.49368 (7)	0.43119 (7)	0.0575 (4)	

### Atomic displacement parameters (Å^2^)

**Table:**

	*U*^11^	*U*^22^	*U*^33^	*U*^12^	*U*^13^	*U*^23^
Ru1A	0.01035 (13)	0.02837 (18)	0.00823 (14)	0.00618 (10)	0.00336 (10)	0.00032 (11)
F1A	0.0101 (9)	0.0323 (13)	0.0186 (11)	0.0008 (8)	0.0049 (8)	−0.0018 (9)
F2A	0.0305 (12)	0.0354 (14)	0.0210 (12)	−0.0015 (10)	0.0187 (10)	0.0000 (10)
F3A	0.0377 (13)	0.0300 (13)	0.0114 (11)	0.0018 (10)	0.0022 (9)	−0.0016 (9)
F4A	0.0178 (11)	0.0308 (13)	0.0239 (12)	−0.0001 (9)	−0.0061 (9)	−0.0040 (10)
F5A	0.0130 (10)	0.0368 (14)	0.0247 (12)	0.0002 (9)	0.0071 (8)	−0.0020 (10)
F6A	0.0294 (12)	0.0373 (15)	0.0261 (13)	−0.0079 (10)	0.0064 (10)	−0.0022 (11)
F7A	0.0391 (15)	0.0571 (19)	0.0183 (12)	−0.0079 (12)	0.0022 (10)	0.0055 (12)
F8A	0.0382 (14)	0.0394 (16)	0.0371 (15)	0.0058 (11)	0.0089 (11)	0.0201 (12)
F9A	0.0625 (18)	0.0239 (14)	0.0397 (16)	0.0080 (12)	0.0136 (13)	0.0035 (12)
F10A	0.0552 (16)	0.0267 (14)	0.0212 (12)	0.0067 (11)	0.0059 (11)	0.0012 (10)
O1A	0.0127 (11)	0.0241 (14)	0.0094 (11)	0.0023 (9)	0.0044 (9)	−0.0003 (10)
O2A	0.0215 (13)	0.0239 (15)	0.0213 (14)	−0.0029 (10)	0.0116 (10)	0.0000 (11)
O3A	0.0133 (11)	0.0254 (14)	0.0159 (12)	0.0065 (9)	0.0027 (9)	0.0002 (10)
O4A	0.0143 (12)	0.0376 (17)	0.0233 (14)	0.0054 (11)	0.0030 (10)	0.0040 (12)
N1A	0.0109 (12)	0.0148 (15)	0.0123 (13)	0.0012 (10)	0.0026 (10)	−0.0007 (11)
N2A	0.0107 (12)	0.0165 (15)	0.0098 (13)	0.0005 (10)	0.0042 (10)	0.0005 (11)
C1A	0.0109 (15)	0.0214 (19)	0.0074 (15)	0.0032 (12)	0.0000 (12)	0.0006 (13)
C2A	0.0127 (15)	0.0204 (19)	0.0163 (17)	−0.0004 (12)	0.0061 (13)	−0.0016 (14)
C3A	0.0119 (15)	0.0200 (19)	0.0148 (16)	0.0030 (12)	0.0063 (12)	−0.0020 (14)
C4A	0.0136 (16)	0.036 (2)	0.0128 (17)	0.0019 (14)	0.0062 (13)	−0.0065 (15)
C5A	0.0149 (16)	0.036 (2)	0.0140 (17)	0.0065 (14)	0.0113 (13)	0.0071 (15)
C6A	0.029 (2)	0.037 (2)	0.0165 (18)	0.0167 (17)	0.0153 (15)	0.0136 (17)
C7A	0.0153 (17)	0.072 (3)	0.0100 (17)	0.0208 (19)	0.0075 (13)	0.0181 (19)
C8A	0.0184 (18)	0.054 (3)	0.0155 (19)	0.0003 (17)	0.0054 (14)	−0.0062 (18)
C9A	0.0201 (18)	0.044 (3)	0.0157 (18)	−0.0023 (16)	0.0080 (14)	−0.0108 (17)
C10A	0.042 (2)	0.032 (3)	0.039 (3)	0.0134 (19)	0.027 (2)	0.015 (2)
C11A	0.032 (2)	0.036 (3)	0.032 (2)	−0.0026 (18)	0.0120 (18)	−0.0132 (19)
C12A	0.055 (3)	0.041 (3)	0.049 (3)	−0.001 (2)	0.026 (3)	−0.012 (2)
C13A	0.040 (3)	0.042 (3)	0.058 (3)	−0.005 (2)	0.018 (2)	−0.010 (3)
C14A	0.0170 (16)	0.0167 (18)	0.0148 (17)	0.0034 (12)	0.0031 (13)	0.0008 (14)
C15A	0.036 (2)	0.022 (2)	0.022 (2)	0.0030 (16)	−0.0018 (16)	0.0001 (16)
C16A	0.050 (3)	0.022 (2)	0.027 (2)	−0.0003 (18)	−0.0020 (19)	−0.0052 (18)
C17A	0.041 (2)	0.020 (2)	0.027 (2)	0.0023 (17)	0.0019 (18)	0.0020 (17)
C18A	0.0148 (16)	0.024 (2)	0.0180 (18)	0.0028 (13)	0.0031 (13)	0.0037 (15)
C19A	0.0127 (15)	0.0229 (19)	0.0126 (16)	−0.0004 (12)	0.0049 (12)	0.0012 (14)
C20A	0.047 (3)	0.027 (2)	0.022 (2)	0.0028 (18)	−0.0021 (18)	−0.0026 (18)
C21A	0.061 (3)	0.020 (2)	0.038 (3)	0.002 (2)	−0.004 (2)	0.003 (2)
C22A	0.0240 (18)	0.025 (2)	0.0144 (17)	−0.0025 (14)	0.0043 (14)	0.0021 (15)
C23A	0.0114 (14)	0.0155 (17)	0.0143 (16)	0.0023 (12)	0.0058 (12)	0.0014 (13)
C24A	0.0120 (15)	0.0201 (19)	0.0154 (17)	0.0019 (12)	0.0046 (12)	−0.0027 (14)
C25A	0.0162 (16)	0.0190 (19)	0.025 (2)	0.0005 (13)	0.0079 (14)	−0.0004 (15)
C26A	0.0180 (17)	0.0202 (19)	0.0219 (19)	0.0045 (13)	0.0104 (14)	0.0040 (15)
C27A	0.0133 (16)	0.026 (2)	0.0158 (17)	0.0046 (13)	0.0028 (13)	0.0032 (15)
C28A	0.0113 (14)	0.0189 (18)	0.0148 (17)	0.0013 (12)	0.0051 (12)	−0.0007 (14)
C29A	0.0158 (16)	0.023 (2)	0.0143 (17)	−0.0019 (13)	0.0024 (13)	−0.0011 (14)
C30A	0.027 (2)	0.020 (2)	0.035 (2)	0.0035 (15)	0.0106 (17)	0.0094 (17)
C31A	0.0243 (18)	0.0173 (19)	0.0218 (19)	0.0019 (14)	−0.0005 (15)	−0.0032 (15)
C32A	0.0133 (15)	0.0178 (18)	0.0129 (16)	0.0040 (12)	0.0056 (12)	0.0017 (13)
C33A	0.0141 (15)	0.0131 (17)	0.0159 (17)	−0.0007 (12)	0.0052 (13)	0.0004 (13)
C34A	0.0138 (15)	0.0164 (18)	0.0182 (17)	0.0022 (12)	0.0053 (13)	0.0000 (14)
C35A	0.0219 (17)	0.0187 (19)	0.0173 (18)	0.0017 (13)	0.0099 (14)	−0.0011 (14)
C36A	0.0298 (19)	0.0135 (18)	0.0111 (16)	0.0011 (13)	0.0049 (14)	0.0002 (13)
C37A	0.0157 (16)	0.0140 (18)	0.0199 (18)	0.0015 (12)	−0.0019 (13)	−0.0015 (14)
C38A	0.0134 (15)	0.0151 (18)	0.0209 (18)	−0.0002 (12)	0.0064 (13)	−0.0003 (14)
C39A	0.0166 (17)	0.024 (2)	0.0188 (18)	0.0065 (14)	0.0057 (13)	0.0010 (15)
C40A	0.0114 (16)	0.034 (2)	0.0186 (18)	0.0060 (14)	0.0023 (13)	0.0048 (16)
C41A	0.0156 (17)	0.032 (2)	0.0221 (19)	−0.0025 (14)	0.0033 (14)	−0.0025 (16)
C42A	0.0173 (17)	0.043 (3)	0.0173 (19)	0.0003 (16)	0.0011 (14)	0.0081 (17)
C43A	0.0233 (19)	0.034 (2)	0.029 (2)	0.0065 (16)	0.0038 (16)	0.0104 (18)
C44A	0.030 (2)	0.023 (2)	0.033 (2)	0.0032 (16)	0.0087 (17)	−0.0002 (18)
C45A	0.028 (2)	0.025 (2)	0.021 (2)	0.0093 (15)	0.0029 (15)	0.0013 (16)
Ru1B	0.00835 (13)	0.01568 (15)	0.00840 (14)	0.00120 (9)	0.00290 (9)	0.00106 (10)
F1B	0.0248 (11)	0.0355 (14)	0.0197 (11)	−0.0034 (9)	0.0042 (9)	−0.0018 (10)
F2B	0.0350 (14)	0.0540 (18)	0.0182 (12)	0.0007 (12)	0.0042 (10)	0.0104 (11)
F3B	0.0428 (15)	0.0350 (15)	0.0442 (16)	0.0099 (11)	0.0181 (12)	0.0252 (13)
F4B	0.0535 (17)	0.0185 (13)	0.0512 (17)	0.0038 (11)	0.0170 (13)	−0.0020 (12)
F5B	0.0452 (15)	0.0299 (14)	0.0225 (12)	0.0023 (11)	0.0087 (10)	−0.0012 (10)
F6B	0.0105 (9)	0.0270 (12)	0.0178 (10)	0.0007 (8)	0.0026 (8)	−0.0021 (9)
F7B	0.0222 (11)	0.0326 (13)	0.0194 (11)	−0.0005 (9)	0.0123 (9)	−0.0013 (9)
F8B	0.0292 (12)	0.0372 (14)	0.0090 (10)	0.0022 (10)	0.0038 (8)	−0.0009 (9)
F9B	0.0164 (11)	0.0481 (16)	0.0174 (11)	0.0018 (9)	−0.0038 (8)	−0.0046 (10)
F10B	0.0110 (10)	0.0540 (16)	0.0182 (11)	0.0022 (9)	0.0049 (8)	−0.0049 (10)
O1B	0.0113 (11)	0.0187 (13)	0.0160 (12)	0.0011 (9)	0.0042 (9)	0.0030 (10)
O2B	0.0097 (11)	0.0285 (15)	0.0254 (14)	0.0000 (10)	0.0033 (10)	0.0086 (11)
O3B	0.0096 (10)	0.0233 (14)	0.0105 (11)	−0.0001 (9)	0.0029 (8)	−0.0008 (10)
O4B	0.0174 (12)	0.0259 (14)	0.0151 (12)	−0.0058 (10)	0.0080 (10)	−0.0003 (11)
N1B	0.0104 (12)	0.0180 (15)	0.0105 (13)	−0.0006 (10)	0.0038 (10)	0.0015 (11)
N2B	0.0095 (12)	0.0173 (15)	0.0095 (13)	−0.0012 (10)	0.0043 (10)	−0.0006 (11)
C1B	0.0106 (14)	0.0175 (18)	0.0092 (15)	0.0001 (12)	0.0010 (11)	−0.0006 (13)
C2B	0.0116 (15)	0.025 (2)	0.0134 (16)	0.0027 (13)	0.0051 (12)	0.0002 (14)
C3B	0.0081 (14)	0.028 (2)	0.0147 (17)	−0.0001 (12)	0.0045 (12)	−0.0026 (14)
C4B	0.0172 (16)	0.028 (2)	0.0117 (17)	0.0072 (14)	0.0059 (13)	0.0070 (14)
C5B	0.0150 (16)	0.033 (2)	0.0097 (17)	0.0027 (14)	0.0026 (13)	0.0026 (14)
C6B	0.0168 (16)	0.027 (2)	0.0112 (16)	0.0017 (13)	0.0062 (13)	−0.0013 (14)
C7B	0.0119 (15)	0.026 (2)	0.0118 (16)	0.0048 (13)	0.0072 (12)	0.0013 (14)
C8B	0.0110 (15)	0.0236 (19)	0.0140 (16)	−0.0001 (12)	0.0067 (12)	0.0034 (14)
C9B	0.0189 (16)	0.0210 (19)	0.0147 (17)	0.0037 (13)	0.0121 (13)	0.0058 (14)
C10B	0.028 (2)	0.021 (2)	0.028 (2)	0.0021 (15)	0.0147 (16)	0.0069 (16)
C11B	0.0253 (19)	0.029 (2)	0.026 (2)	−0.0011 (16)	0.0085 (16)	−0.0091 (17)
C12B	0.044 (3)	0.029 (3)	0.044 (3)	0.0033 (19)	0.012 (2)	−0.006 (2)
C13B	0.071 (4)	0.048 (3)	0.041 (3)	0.014 (3)	−0.012 (3)	−0.018 (3)
C14B	0.0138 (15)	0.0167 (18)	0.0136 (16)	0.0035 (12)	0.0044 (12)	0.0031 (13)
C15B	0.0104 (14)	0.024 (2)	0.0181 (18)	0.0039 (13)	0.0055 (12)	0.0009 (14)
C16B	0.0158 (16)	0.029 (2)	0.0178 (18)	0.0107 (14)	0.0037 (13)	0.0076 (15)
C17B	0.0241 (18)	0.022 (2)	0.029 (2)	0.0103 (15)	0.0125 (16)	0.0115 (17)
C18B	0.0200 (18)	0.019 (2)	0.029 (2)	0.0032 (14)	0.0064 (15)	0.0030 (16)
C19B	0.0114 (15)	0.0185 (19)	0.0219 (18)	0.0032 (12)	0.0048 (13)	0.0006 (14)
C20B	0.0146 (16)	0.028 (2)	0.0147 (17)	−0.0006 (13)	0.0011 (13)	−0.0024 (15)
C21B	0.032 (2)	0.033 (3)	0.042 (3)	0.0123 (18)	0.0145 (19)	0.019 (2)
C22B	0.0243 (19)	0.019 (2)	0.023 (2)	−0.0008 (14)	−0.0016 (15)	−0.0023 (16)
C23B	0.0101 (14)	0.0152 (17)	0.0164 (17)	−0.0024 (11)	0.0056 (12)	0.0010 (13)
C24B	0.0158 (16)	0.024 (2)	0.0159 (17)	−0.0022 (13)	0.0056 (13)	−0.0030 (14)
C25B	0.0228 (18)	0.021 (2)	0.024 (2)	−0.0050 (14)	0.0072 (15)	−0.0056 (16)
C26B	0.0180 (17)	0.021 (2)	0.027 (2)	−0.0037 (13)	0.0075 (15)	−0.0027 (16)
C27B	0.0138 (16)	0.0205 (19)	0.0176 (17)	−0.0008 (13)	0.0041 (13)	0.0031 (14)
C28B	0.0086 (14)	0.0230 (19)	0.0158 (17)	−0.0021 (12)	0.0047 (12)	−0.0006 (14)
C29B	0.034 (2)	0.032 (2)	0.0136 (18)	−0.0062 (17)	−0.0001 (15)	−0.0061 (16)
C30B	0.035 (2)	0.023 (2)	0.037 (2)	−0.0022 (17)	0.0069 (19)	−0.0013 (19)
C31B	0.0171 (16)	0.022 (2)	0.0144 (17)	−0.0005 (13)	0.0043 (13)	−0.0003 (14)
C32B	0.0134 (15)	0.0216 (19)	0.0150 (17)	0.0034 (13)	0.0062 (12)	0.0035 (14)
C33B	0.0097 (15)	0.023 (2)	0.0216 (18)	0.0044 (12)	0.0054 (13)	0.0074 (15)
C34B	0.0107 (15)	0.027 (2)	0.0221 (19)	0.0012 (13)	0.0040 (13)	0.0020 (15)
C35B	0.0161 (17)	0.046 (3)	0.0146 (18)	0.0027 (16)	0.0038 (14)	0.0064 (17)
C36B	0.0217 (19)	0.027 (2)	0.034 (2)	0.0065 (15)	0.0092 (16)	0.0126 (18)
C37B	0.028 (2)	0.024 (2)	0.032 (2)	0.0035 (15)	0.0100 (17)	0.0048 (17)
C38B	0.0206 (18)	0.023 (2)	0.024 (2)	0.0041 (14)	0.0074 (14)	0.0059 (16)
C39B	0.0142 (15)	0.0162 (17)	0.0114 (16)	0.0027 (12)	0.0017 (12)	−0.0017 (13)
C40B	0.0117 (15)	0.0158 (17)	0.0128 (16)	−0.0007 (12)	0.0027 (12)	−0.0031 (13)
C41B	0.0126 (15)	0.0141 (17)	0.0149 (17)	−0.0004 (12)	0.0023 (12)	−0.0005 (13)
C42B	0.0173 (16)	0.0170 (18)	0.0168 (17)	−0.0004 (12)	0.0105 (13)	−0.0004 (14)
C43B	0.0227 (18)	0.0154 (18)	0.0112 (16)	−0.0006 (13)	0.0021 (13)	−0.0025 (13)
C44B	0.0127 (16)	0.025 (2)	0.0147 (17)	0.0001 (13)	−0.0024 (13)	−0.0027 (14)
C45B	0.0137 (16)	0.0206 (19)	0.0170 (18)	0.0014 (12)	0.0044 (13)	−0.0031 (14)
C1X	0.0226 (19)	0.035 (2)	0.023 (2)	−0.0010 (16)	0.0071 (15)	−0.0024 (17)
Cl1	0.0368 (6)	0.0338 (6)	0.0406 (6)	−0.0042 (4)	0.0062 (5)	0.0053 (5)
Cl2	0.0271 (5)	0.0311 (6)	0.0569 (7)	−0.0047 (4)	0.0086 (5)	0.0031 (5)
C2X	0.0212 (18)	0.039 (3)	0.021 (2)	0.0028 (16)	0.0077 (15)	0.0008 (17)
Cl3	0.0463 (7)	0.0596 (9)	0.0342 (6)	−0.0093 (6)	0.0024 (5)	0.0200 (6)
Cl4	0.0283 (6)	0.0371 (7)	0.1310 (15)	−0.0020 (5)	0.0101 (7)	0.0336 (8)
C3X	0.036 (3)	0.044 (3)	0.059 (3)	0.012 (2)	0.017 (2)	0.013 (3)
Cl5	0.0415 (6)	0.0417 (7)	0.0538 (8)	−0.0024 (5)	0.0073 (5)	0.0083 (6)
Cl6	0.0362 (6)	0.0881 (11)	0.0528 (8)	0.0171 (6)	0.0149 (6)	0.0301 (8)
C4X	0.030 (2)	0.027 (2)	0.055 (3)	−0.0020 (17)	0.016 (2)	−0.009 (2)
Cl7	0.0338 (6)	0.0398 (7)	0.0441 (7)	0.0068 (4)	0.0134 (5)	0.0000 (5)
Cl8	0.0607 (8)	0.0530 (9)	0.0687 (10)	−0.0192 (6)	0.0346 (7)	−0.0281 (7)

### Geometric parameters (Å, º)

**Table:**

Ru1A—O1A	2.103 (2)	Ru1B—C9B	2.250 (3)
Ru1A—O3A	2.109 (3)	F1B—C34B	1.339 (5)
Ru1A—C1A	2.116 (3)	F2B—C35B	1.336 (4)
Ru1A—C7A	2.182 (4)	F3B—C36B	1.349 (5)
Ru1A—C8A	2.196 (4)	F4B—C37B	1.335 (5)
Ru1A—C6A	2.218 (4)	F5B—C38B	1.331 (5)
Ru1A—C4A	2.220 (3)	F6B—C41B	1.337 (4)
Ru1A—C9A	2.221 (4)	F7B—C42B	1.333 (4)
Ru1A—C5A	2.228 (3)	F8B—C43B	1.339 (4)
F1A—C34A	1.336 (4)	F9B—C44B	1.341 (4)
F2A—C35A	1.336 (4)	F10B—C45B	1.339 (4)
F3A—C36A	1.336 (4)	O1B—C32B	1.274 (4)
F4A—C37A	1.343 (4)	O2B—C32B	1.234 (4)
F5A—C38A	1.338 (4)	O3B—C39B	1.267 (4)
F6A—C41A	1.335 (5)	O4B—C39B	1.229 (4)
F7A—C42A	1.335 (5)	N1B—C1B	1.366 (4)
F8A—C43A	1.331 (5)	N1B—C2B	1.385 (4)
F9A—C44A	1.333 (5)	N1B—C14B	1.440 (4)
F10A—C45A	1.329 (5)	N2B—C1B	1.369 (4)
O1A—C32A	1.272 (4)	N2B—C3B	1.394 (4)
O2A—C32A	1.224 (4)	N2B—C23B	1.443 (4)
O3A—C39A	1.273 (4)	C2B—C3B	1.329 (5)
O4A—C39A	1.223 (5)	C2B—H2B	0.9500
N1A—C1A	1.372 (4)	C3B—H3B	0.9500
N1A—C2A	1.384 (4)	C4B—C9B	1.401 (5)
N1A—C14A	1.448 (5)	C4B—C5B	1.415 (6)
N2A—C1A	1.365 (4)	C4B—H4B	0.9500
N2A—C3A	1.393 (4)	C5B—C6B	1.418 (5)
N2A—C23A	1.449 (4)	C5B—H5B	0.9500
C2A—C3A	1.337 (5)	C6B—C7B	1.424 (5)
C2A—H2A	0.9500	C6B—C11B	1.503 (5)
C3A—H3A	0.9500	C7B—C8B	1.389 (5)
C4A—C5A	1.382 (6)	C7B—H7B	0.9500
C4A—C9A	1.442 (5)	C8B—C9B	1.429 (5)
C4A—H4A	0.9500	C8B—H8B	0.9500
C5A—C6A	1.428 (5)	C9B—C10B	1.494 (5)
C5A—H5A	0.9500	C10B—H10D	0.9800
C6A—C7A	1.435 (6)	C10B—H10E	0.9800
C6A—C10A	1.479 (6)	C10B—H10F	0.9800
C7A—C8A	1.404 (7)	C11B—C12B	1.512 (6)
C7A—H7A	0.9500	C11B—C13B	1.542 (7)
C8A—C9A	1.400 (6)	C11B—H11B	1.0000
C8A—H8A	0.9500	C12B—H12D	0.9800
C9A—C11A	1.531 (6)	C12B—H12E	0.9800
C10A—H10A	0.9800	C12B—H12F	0.9800
C10A—H10B	0.9800	C13B—H13D	0.9800
C10A—H10C	0.9800	C13B—H13E	0.9800
C11A—C13A	1.511 (6)	C13B—H13F	0.9800
C11A—C12A	1.526 (6)	C14B—C19B	1.397 (5)
C11A—H11A	1.0000	C14B—C15B	1.399 (5)
C12A—H12A	0.9800	C15B—C16B	1.391 (5)
C12A—H12B	0.9800	C15B—C20B	1.499 (5)
C12A—H12C	0.9800	C16B—C17B	1.394 (6)
C13A—H13A	0.9800	C16B—H16B	0.9500
C13A—H13B	0.9800	C17B—C18B	1.381 (6)
C13A—H13C	0.9800	C17B—C21B	1.501 (5)
C14A—C15A	1.389 (5)	C18B—C19B	1.392 (5)
C14A—C19A	1.393 (5)	C18B—H18B	0.9500
C15A—C16A	1.393 (6)	C19B—C22B	1.498 (5)
C15A—C20A	1.501 (6)	C20B—H20D	0.9800
C16A—C17A	1.376 (6)	C20B—H20E	0.9800
C16A—H16A	0.9500	C20B—H20F	0.9800
C17A—C18A	1.383 (6)	C21B—H21D	0.9800
C17A—C21A	1.503 (6)	C21B—H21E	0.9800
C18A—C19A	1.383 (5)	C21B—H21F	0.9800
C18A—H18A	0.9500	C22B—H22D	0.9800
C19A—C22A	1.495 (5)	C22B—H22E	0.9800
C20A—H20A	0.9800	C22B—H22F	0.9800
C20A—H20B	0.9800	C23B—C24B	1.385 (5)
C20A—H20C	0.9800	C23B—C28B	1.393 (5)
C21A—H21A	0.9800	C24B—C25B	1.387 (6)
C21A—H21B	0.9800	C24B—C29B	1.507 (5)
C21A—H21C	0.9800	C25B—C26B	1.380 (6)
C22A—H22A	0.9800	C25B—H25B	0.9500
C22A—H22B	0.9800	C26B—C27B	1.395 (5)
C22A—H22C	0.9800	C26B—C30B	1.508 (6)
C23A—C24A	1.383 (5)	C27B—C28B	1.390 (5)
C23A—C28A	1.400 (5)	C27B—H27B	0.9500
C24A—C25A	1.396 (5)	C28B—C31B	1.498 (5)
C24A—C31A	1.496 (5)	C29B—H29D	0.9800
C25A—C26A	1.382 (5)	C29B—H29E	0.9800
C25A—H25A	0.9500	C29B—H29F	0.9800
C26A—C27A	1.399 (5)	C30B—H30D	0.9800
C26A—C30A	1.495 (5)	C30B—H30E	0.9800
C27A—C28A	1.390 (5)	C30B—H30F	0.9800
C27A—H27A	0.9500	C31B—H31D	0.9800
C28A—C29A	1.497 (5)	C31B—H31E	0.9800
C29A—H29A	0.9800	C31B—H31F	0.9800
C29A—H29B	0.9800	C32B—C33B	1.523 (5)
C29A—H29C	0.9800	C33B—C34B	1.374 (5)
C30A—H30A	0.9800	C33B—C38B	1.387 (6)
C30A—H30B	0.9800	C34B—C35B	1.384 (6)
C30A—H30C	0.9800	C35B—C36B	1.368 (6)
C31A—H31A	0.9800	C36B—C37B	1.363 (6)
C31A—H31B	0.9800	C37B—C38B	1.383 (6)
C31A—H31C	0.9800	C39B—C40B	1.520 (5)
C32A—C33A	1.521 (5)	C40B—C45B	1.391 (5)
C33A—C38A	1.389 (5)	C40B—C41B	1.396 (5)
C33A—C34A	1.397 (5)	C41B—C42B	1.381 (5)
C34A—C35A	1.378 (5)	C42B—C43B	1.377 (5)
C35A—C36A	1.378 (5)	C43B—C44B	1.363 (5)
C36A—C37A	1.373 (5)	C44B—C45B	1.383 (5)
C37A—C38A	1.380 (5)	C1X—Cl1	1.759 (4)
C39A—C40A	1.543 (5)	C1X—Cl2	1.771 (4)
C40A—C41A	1.374 (5)	C1X—H1X1	0.9900
C40A—C45A	1.378 (6)	C1X—H1X2	0.9900
C41A—C42A	1.388 (6)	C2X—Cl3	1.742 (4)
C42A—C43A	1.377 (6)	C2X—Cl4	1.760 (5)
C43A—C44A	1.372 (6)	C2X—H2X1	0.9900
C44A—C45A	1.396 (6)	C2X—H2X2	0.9900
Ru1B—O3B	2.101 (2)	C3X—Cl5	1.744 (5)
Ru1B—O1B	2.107 (2)	C3X—Cl6	1.789 (6)
Ru1B—C1B	2.122 (3)	C3X—H3X1	0.9900
Ru1B—C5B	2.176 (4)	C3X—H3X2	0.9900
Ru1B—C7B	2.209 (3)	C4X—Cl8	1.770 (5)
Ru1B—C8B	2.209 (3)	C4X—Cl7	1.776 (5)
Ru1B—C4B	2.219 (3)	C4X—H4X1	0.9900
Ru1B—C6B	2.220 (3)	C4X—H4X2	0.9900
			
O1A—Ru1A—O3A	78.67 (10)	C1B—Ru1B—C8B	157.82 (13)
O1A—Ru1A—C1A	78.32 (11)	C5B—Ru1B—C8B	79.21 (13)
O3A—Ru1A—C1A	88.46 (11)	C7B—Ru1B—C8B	36.65 (14)
O1A—Ru1A—C7A	129.89 (16)	O3B—Ru1B—C4B	124.52 (12)
O3A—Ru1A—C7A	151.38 (16)	O1B—Ru1B—C4B	157.31 (12)
C1A—Ru1A—C7A	95.31 (13)	C1B—Ru1B—C4B	97.37 (13)
O1A—Ru1A—C8A	167.02 (14)	C5B—Ru1B—C4B	37.54 (14)
O3A—Ru1A—C8A	113.98 (15)	C7B—Ru1B—C4B	78.55 (13)
C1A—Ru1A—C8A	98.46 (13)	C8B—Ru1B—C4B	66.36 (13)
C7A—Ru1A—C8A	37.40 (18)	O3B—Ru1B—C6B	156.81 (11)
O1A—Ru1A—C6A	101.24 (13)	O1B—Ru1B—C6B	90.97 (12)
O3A—Ru1A—C6A	154.25 (12)	C1B—Ru1B—C6B	122.03 (13)
C1A—Ru1A—C6A	116.94 (13)	C5B—Ru1B—C6B	37.62 (13)
C7A—Ru1A—C6A	38.06 (16)	C7B—Ru1B—C6B	37.51 (12)
C8A—Ru1A—C6A	68.78 (17)	C8B—Ru1B—C6B	67.33 (13)
O1A—Ru1A—C4A	117.80 (11)	C4B—Ru1B—C6B	67.64 (14)
O3A—Ru1A—C4A	90.37 (12)	O3B—Ru1B—C9B	99.37 (12)
C1A—Ru1A—C4A	163.24 (13)	O1B—Ru1B—C9B	150.65 (11)
C7A—Ru1A—C4A	78.00 (14)	C1B—Ru1B—C9B	121.03 (13)
C8A—Ru1A—C4A	66.86 (14)	C5B—Ru1B—C9B	67.23 (14)
C6A—Ru1A—C4A	66.63 (14)	C7B—Ru1B—C9B	66.84 (13)
O1A—Ru1A—C9A	152.72 (12)	C8B—Ru1B—C9B	37.38 (12)
O3A—Ru1A—C9A	87.87 (13)	C4B—Ru1B—C9B	36.55 (13)
C1A—Ru1A—C9A	125.35 (14)	C6B—Ru1B—C9B	80.09 (14)
C7A—Ru1A—C9A	66.86 (17)	C32B—O1B—Ru1B	120.6 (2)
C8A—Ru1A—C9A	36.95 (15)	C39B—O3B—Ru1B	127.4 (2)
C6A—Ru1A—C9A	80.86 (16)	C1B—N1B—C2B	111.7 (3)
C4A—Ru1A—C9A	37.89 (13)	C1B—N1B—C14B	128.6 (3)
O1A—Ru1A—C5A	97.34 (12)	C2B—N1B—C14B	118.7 (3)
O3A—Ru1A—C5A	116.79 (12)	C1B—N2B—C3B	111.5 (3)
C1A—Ru1A—C5A	153.30 (14)	C1B—N2B—C23B	130.0 (3)
C7A—Ru1A—C5A	67.05 (14)	C3B—N2B—C23B	117.9 (3)
C8A—Ru1A—C5A	79.83 (15)	N1B—C1B—N2B	102.9 (3)
C6A—Ru1A—C5A	37.45 (13)	N1B—C1B—Ru1B	126.7 (2)
C4A—Ru1A—C5A	36.21 (15)	N2B—C1B—Ru1B	129.4 (2)
C9A—Ru1A—C5A	67.65 (15)	C3B—C2B—N1B	107.3 (3)
C32A—O1A—Ru1A	128.3 (2)	C3B—C2B—H2B	126.4
C39A—O3A—Ru1A	121.1 (2)	N1B—C2B—H2B	126.4
C1A—N1A—C2A	111.9 (3)	C2B—C3B—N2B	106.6 (3)
C1A—N1A—C14A	130.6 (3)	C2B—C3B—H3B	126.7
C2A—N1A—C14A	117.0 (3)	N2B—C3B—H3B	126.7
C1A—N2A—C3A	111.7 (3)	C9B—C4B—C5B	121.0 (3)
C1A—N2A—C23A	130.3 (3)	C9B—C4B—Ru1B	72.9 (2)
C3A—N2A—C23A	117.0 (3)	C5B—C4B—Ru1B	69.6 (2)
N2A—C1A—N1A	102.8 (3)	C9B—C4B—H4B	119.5
N2A—C1A—Ru1A	126.8 (2)	C5B—C4B—H4B	119.5
N1A—C1A—Ru1A	129.1 (2)	Ru1B—C4B—H4B	130.7
C3A—C2A—N1A	106.9 (3)	C4B—C5B—C6B	121.4 (3)
C3A—C2A—H2A	126.6	C4B—C5B—Ru1B	72.9 (2)
N1A—C2A—H2A	126.6	C6B—C5B—Ru1B	72.9 (2)
C2A—C3A—N2A	106.7 (3)	C4B—C5B—H5B	119.3
C2A—C3A—H3A	126.6	C6B—C5B—H5B	119.3
N2A—C3A—H3A	126.6	Ru1B—C5B—H5B	127.0
C5A—C4A—C9A	122.5 (4)	C5B—C6B—C7B	117.0 (3)
C5A—C4A—Ru1A	72.2 (2)	C5B—C6B—C11B	120.8 (3)
C9A—C4A—Ru1A	71.1 (2)	C7B—C6B—C11B	122.2 (3)
C5A—C4A—H4A	118.7	C5B—C6B—Ru1B	69.5 (2)
C9A—C4A—H4A	118.7	C7B—C6B—Ru1B	70.81 (19)
Ru1A—C4A—H4A	130.9	C11B—C6B—Ru1B	132.3 (3)
C4A—C5A—C6A	120.3 (4)	C8B—C7B—C6B	121.6 (3)
C4A—C5A—Ru1A	71.6 (2)	C8B—C7B—Ru1B	71.69 (19)
C6A—C5A—Ru1A	70.9 (2)	C6B—C7B—Ru1B	71.68 (19)
C4A—C5A—H5A	119.8	C8B—C7B—H7B	119.2
C6A—C5A—H5A	119.8	C6B—C7B—H7B	119.2
Ru1A—C5A—H5A	130.3	Ru1B—C7B—H7B	130.1
C5A—C6A—C7A	116.6 (4)	C7B—C8B—C9B	121.2 (3)
C5A—C6A—C10A	120.0 (4)	C7B—C8B—Ru1B	71.66 (19)
C7A—C6A—C10A	123.3 (4)	C9B—C8B—Ru1B	72.85 (19)
C5A—C6A—Ru1A	71.6 (2)	C7B—C8B—H8B	119.4
C7A—C6A—Ru1A	69.6 (2)	C9B—C8B—H8B	119.4
C10A—C6A—Ru1A	132.2 (3)	Ru1B—C8B—H8B	128.4
C8A—C7A—C6A	122.9 (3)	C4B—C9B—C8B	117.7 (3)
C8A—C7A—Ru1A	71.8 (2)	C4B—C9B—C10B	121.4 (3)
C6A—C7A—Ru1A	72.3 (2)	C8B—C9B—C10B	120.8 (3)
C8A—C7A—H7A	118.6	C4B—C9B—Ru1B	70.5 (2)
C6A—C7A—H7A	118.6	C8B—C9B—Ru1B	69.77 (19)
Ru1A—C7A—H7A	130.0	C10B—C9B—Ru1B	133.8 (3)
C9A—C8A—C7A	119.8 (4)	C9B—C10B—H10D	109.5
C9A—C8A—Ru1A	72.5 (2)	C9B—C10B—H10E	109.5
C7A—C8A—Ru1A	70.8 (2)	H10D—C10B—H10E	109.5
C9A—C8A—H8A	120.1	C9B—C10B—H10F	109.5
C7A—C8A—H8A	120.1	H10D—C10B—H10F	109.5
Ru1A—C8A—H8A	128.9	H10E—C10B—H10F	109.5
C8A—C9A—C4A	117.8 (4)	C6B—C11B—C12B	113.5 (3)
C8A—C9A—C11A	124.8 (4)	C6B—C11B—C13B	108.8 (4)
C4A—C9A—C11A	117.4 (4)	C12B—C11B—C13B	107.8 (4)
C8A—C9A—Ru1A	70.5 (2)	C6B—C11B—H11B	108.9
C4A—C9A—Ru1A	71.0 (2)	C12B—C11B—H11B	108.9
C11A—C9A—Ru1A	131.5 (3)	C13B—C11B—H11B	108.9
C6A—C10A—H10A	109.5	C11B—C12B—H12D	109.5
C6A—C10A—H10B	109.5	C11B—C12B—H12E	109.5
H10A—C10A—H10B	109.5	H12D—C12B—H12E	109.5
C6A—C10A—H10C	109.5	C11B—C12B—H12F	109.5
H10A—C10A—H10C	109.5	H12D—C12B—H12F	109.5
H10B—C10A—H10C	109.5	H12E—C12B—H12F	109.5
C13A—C11A—C12A	111.2 (4)	C11B—C13B—H13D	109.5
C13A—C11A—C9A	116.4 (4)	C11B—C13B—H13E	109.5
C12A—C11A—C9A	107.4 (4)	H13D—C13B—H13E	109.5
C13A—C11A—H11A	107.2	C11B—C13B—H13F	109.5
C12A—C11A—H11A	107.2	H13D—C13B—H13F	109.5
C9A—C11A—H11A	107.2	H13E—C13B—H13F	109.5
C11A—C12A—H12A	109.5	C19B—C14B—C15B	121.7 (3)
C11A—C12A—H12B	109.5	C19B—C14B—N1B	120.1 (3)
H12A—C12A—H12B	109.5	C15B—C14B—N1B	117.8 (3)
C11A—C12A—H12C	109.5	C16B—C15B—C14B	118.1 (3)
H12A—C12A—H12C	109.5	C16B—C15B—C20B	120.5 (3)
H12B—C12A—H12C	109.5	C14B—C15B—C20B	121.5 (3)
C11A—C13A—H13A	109.5	C15B—C16B—C17B	121.7 (3)
C11A—C13A—H13B	109.5	C15B—C16B—H16B	119.1
H13A—C13A—H13B	109.5	C17B—C16B—H16B	119.1
C11A—C13A—H13C	109.5	C18B—C17B—C16B	118.2 (4)
H13A—C13A—H13C	109.5	C18B—C17B—C21B	122.0 (4)
H13B—C13A—H13C	109.5	C16B—C17B—C21B	119.8 (4)
C15A—C14A—C19A	121.7 (4)	C17B—C18B—C19B	122.5 (4)
C15A—C14A—N1A	118.5 (3)	C17B—C18B—H18B	118.8
C19A—C14A—N1A	118.7 (3)	C19B—C18B—H18B	118.8
C14A—C15A—C16A	117.8 (4)	C18B—C19B—C14B	117.6 (3)
C14A—C15A—C20A	121.2 (4)	C18B—C19B—C22B	120.6 (3)
C16A—C15A—C20A	121.0 (4)	C14B—C19B—C22B	121.7 (3)
C17A—C16A—C15A	122.2 (4)	C15B—C20B—H20D	109.5
C17A—C16A—H16A	118.9	C15B—C20B—H20E	109.5
C15A—C16A—H16A	118.9	H20D—C20B—H20E	109.5
C16A—C17A—C18A	118.0 (4)	C15B—C20B—H20F	109.5
C16A—C17A—C21A	120.8 (4)	H20D—C20B—H20F	109.5
C18A—C17A—C21A	121.2 (4)	H20E—C20B—H20F	109.5
C19A—C18A—C17A	122.5 (3)	C17B—C21B—H21D	109.5
C19A—C18A—H18A	118.7	C17B—C21B—H21E	109.5
C17A—C18A—H18A	118.7	H21D—C21B—H21E	109.5
C18A—C19A—C14A	117.6 (3)	C17B—C21B—H21F	109.5
C18A—C19A—C22A	121.3 (3)	H21D—C21B—H21F	109.5
C14A—C19A—C22A	121.1 (3)	H21E—C21B—H21F	109.5
C15A—C20A—H20A	109.5	C19B—C22B—H22D	109.5
C15A—C20A—H20B	109.5	C19B—C22B—H22E	109.5
H20A—C20A—H20B	109.5	H22D—C22B—H22E	109.5
C15A—C20A—H20C	109.5	C19B—C22B—H22F	109.5
H20A—C20A—H20C	109.5	H22D—C22B—H22F	109.5
H20B—C20A—H20C	109.5	H22E—C22B—H22F	109.5
C17A—C21A—H21A	109.5	C24B—C23B—C28B	122.4 (3)
C17A—C21A—H21B	109.5	C24B—C23B—N2B	118.4 (3)
H21A—C21A—H21B	109.5	C28B—C23B—N2B	118.9 (3)
C17A—C21A—H21C	109.5	C23B—C24B—C25B	118.1 (3)
H21A—C21A—H21C	109.5	C23B—C24B—C29B	121.5 (3)
H21B—C21A—H21C	109.5	C25B—C24B—C29B	120.4 (3)
C19A—C22A—H22A	109.5	C26B—C25B—C24B	121.9 (4)
C19A—C22A—H22B	109.5	C26B—C25B—H25B	119.0
H22A—C22A—H22B	109.5	C24B—C25B—H25B	119.0
C19A—C22A—H22C	109.5	C25B—C26B—C27B	118.2 (4)
H22A—C22A—H22C	109.5	C25B—C26B—C30B	120.1 (4)
H22B—C22A—H22C	109.5	C27B—C26B—C30B	121.7 (4)
C24A—C23A—C28A	122.3 (3)	C28B—C27B—C26B	122.0 (3)
C24A—C23A—N2A	119.6 (3)	C28B—C27B—H27B	119.0
C28A—C23A—N2A	117.4 (3)	C26B—C27B—H27B	119.0
C23A—C24A—C25A	117.6 (3)	C27B—C28B—C23B	117.3 (3)
C23A—C24A—C31A	122.1 (3)	C27B—C28B—C31B	121.2 (3)
C25A—C24A—C31A	120.2 (3)	C23B—C28B—C31B	121.4 (3)
C26A—C25A—C24A	122.3 (4)	C24B—C29B—H29D	109.5
C26A—C25A—H25A	118.8	C24B—C29B—H29E	109.5
C24A—C25A—H25A	118.8	H29D—C29B—H29E	109.5
C25A—C26A—C27A	118.3 (3)	C24B—C29B—H29F	109.5
C25A—C26A—C30A	121.9 (4)	H29D—C29B—H29F	109.5
C27A—C26A—C30A	119.8 (3)	H29E—C29B—H29F	109.5
C28A—C27A—C26A	121.4 (3)	C26B—C30B—H30D	109.5
C28A—C27A—H27A	119.3	C26B—C30B—H30E	109.5
C26A—C27A—H27A	119.3	H30D—C30B—H30E	109.5
C27A—C28A—C23A	118.0 (3)	C26B—C30B—H30F	109.5
C27A—C28A—C29A	119.9 (3)	H30D—C30B—H30F	109.5
C23A—C28A—C29A	122.1 (3)	H30E—C30B—H30F	109.5
C28A—C29A—H29A	109.5	C28B—C31B—H31D	109.5
C28A—C29A—H29B	109.5	C28B—C31B—H31E	109.5
H29A—C29A—H29B	109.5	H31D—C31B—H31E	109.5
C28A—C29A—H29C	109.5	C28B—C31B—H31F	109.5
H29A—C29A—H29C	109.5	H31D—C31B—H31F	109.5
H29B—C29A—H29C	109.5	H31E—C31B—H31F	109.5
C26A—C30A—H30A	109.5	O2B—C32B—O1B	129.1 (3)
C26A—C30A—H30B	109.5	O2B—C32B—C33B	117.3 (3)
H30A—C30A—H30B	109.5	O1B—C32B—C33B	113.5 (3)
C26A—C30A—H30C	109.5	C34B—C33B—C38B	116.7 (3)
H30A—C30A—H30C	109.5	C34B—C33B—C32B	121.0 (3)
H30B—C30A—H30C	109.5	C38B—C33B—C32B	122.2 (3)
C24A—C31A—H31A	109.5	F1B—C34B—C33B	119.9 (3)
C24A—C31A—H31B	109.5	F1B—C34B—C35B	117.8 (3)
H31A—C31A—H31B	109.5	C33B—C34B—C35B	122.3 (4)
C24A—C31A—H31C	109.5	F2B—C35B—C36B	120.0 (4)
H31A—C31A—H31C	109.5	F2B—C35B—C34B	120.8 (4)
H31B—C31A—H31C	109.5	C36B—C35B—C34B	119.2 (4)
O2A—C32A—O1A	128.5 (3)	F3B—C36B—C37B	119.2 (4)
O2A—C32A—C33A	118.8 (3)	F3B—C36B—C35B	120.3 (4)
O1A—C32A—C33A	112.7 (3)	C37B—C36B—C35B	120.5 (4)
C38A—C33A—C34A	115.5 (3)	F4B—C37B—C36B	120.2 (4)
C38A—C33A—C32A	121.3 (3)	F4B—C37B—C38B	120.3 (4)
C34A—C33A—C32A	123.2 (3)	C36B—C37B—C38B	119.4 (4)
F1A—C34A—C35A	115.9 (3)	F5B—C38B—C37B	117.5 (4)
F1A—C34A—C33A	121.2 (3)	F5B—C38B—C33B	120.6 (3)
C35A—C34A—C33A	122.8 (3)	C37B—C38B—C33B	121.9 (4)
F2A—C35A—C36A	120.0 (3)	O4B—C39B—O3B	129.2 (3)
F2A—C35A—C34A	120.5 (3)	O4B—C39B—C40B	117.6 (3)
C36A—C35A—C34A	119.5 (3)	O3B—C39B—C40B	113.1 (3)
F3A—C36A—C37A	120.8 (3)	C45B—C40B—C41B	115.7 (3)
F3A—C36A—C35A	119.7 (3)	C45B—C40B—C39B	121.3 (3)
C37A—C36A—C35A	119.5 (3)	C41B—C40B—C39B	123.0 (3)
F4A—C37A—C36A	119.3 (3)	F6B—C41B—C42B	116.3 (3)
F4A—C37A—C38A	120.5 (3)	F6B—C41B—C40B	121.1 (3)
C36A—C37A—C38A	120.2 (3)	C42B—C41B—C40B	122.6 (3)
F5A—C38A—C37A	116.3 (3)	F7B—C42B—C43B	120.1 (3)
F5A—C38A—C33A	121.3 (3)	F7B—C42B—C41B	120.6 (3)
C37A—C38A—C33A	122.4 (3)	C43B—C42B—C41B	119.3 (3)
O4A—C39A—O3A	129.8 (4)	F8B—C43B—C44B	120.4 (3)
O4A—C39A—C40A	118.2 (3)	F8B—C43B—C42B	119.5 (3)
O3A—C39A—C40A	112.0 (3)	C44B—C43B—C42B	120.1 (3)
C41A—C40A—C45A	117.3 (4)	F9B—C44B—C43B	119.9 (3)
C41A—C40A—C39A	121.3 (4)	F9B—C44B—C45B	120.2 (3)
C45A—C40A—C39A	121.4 (3)	C43B—C44B—C45B	120.0 (3)
F6A—C41A—C40A	119.9 (4)	F10B—C45B—C44B	117.1 (3)
F6A—C41A—C42A	117.9 (4)	F10B—C45B—C40B	120.6 (3)
C40A—C41A—C42A	122.2 (4)	C44B—C45B—C40B	122.3 (3)
F7A—C42A—C43A	120.2 (4)	Cl1—C1X—Cl2	111.0 (2)
F7A—C42A—C41A	120.5 (4)	Cl1—C1X—H1X1	109.4
C43A—C42A—C41A	119.3 (4)	Cl2—C1X—H1X1	109.4
F8A—C43A—C44A	119.6 (4)	Cl1—C1X—H1X2	109.4
F8A—C43A—C42A	120.2 (4)	Cl2—C1X—H1X2	109.4
C44A—C43A—C42A	120.1 (4)	H1X1—C1X—H1X2	108.0
F9A—C44A—C43A	120.5 (4)	Cl3—C2X—Cl4	109.9 (2)
F9A—C44A—C45A	120.3 (4)	Cl3—C2X—H2X1	109.7
C43A—C44A—C45A	119.2 (4)	Cl4—C2X—H2X1	109.7
F10A—C45A—C40A	121.0 (4)	Cl3—C2X—H2X2	109.7
F10A—C45A—C44A	117.2 (4)	Cl4—C2X—H2X2	109.7
C40A—C45A—C44A	121.9 (4)	H2X1—C2X—H2X2	108.2
O3B—Ru1B—O1B	78.16 (10)	Cl5—C3X—Cl6	110.2 (3)
O3B—Ru1B—C1B	78.35 (11)	Cl5—C3X—H3X1	109.6
O1B—Ru1B—C1B	87.44 (11)	Cl6—C3X—H3X1	109.6
O3B—Ru1B—C5B	161.39 (12)	Cl5—C3X—H3X2	109.6
O1B—Ru1B—C5B	119.95 (13)	Cl6—C3X—H3X2	109.6
C1B—Ru1B—C5B	97.15 (13)	H3X1—C3X—H3X2	108.1
O3B—Ru1B—C7B	120.91 (11)	Cl8—C4X—Cl7	110.4 (2)
O1B—Ru1B—C7B	89.09 (11)	Cl8—C4X—H4X1	109.6
C1B—Ru1B—C7B	159.19 (13)	Cl7—C4X—H4X1	109.6
C5B—Ru1B—C7B	67.09 (13)	Cl8—C4X—H4X2	109.6
O3B—Ru1B—C8B	98.07 (11)	Cl7—C4X—H4X2	109.6
O1B—Ru1B—C8B	113.48 (11)	H4X1—C4X—H4X2	108.1
			
O3A—Ru1A—O1A—C32A	111.5 (3)	O3B—Ru1B—O1B—C32B	−63.1 (3)
C1A—Ru1A—O1A—C32A	−157.8 (3)	C1B—Ru1B—O1B—C32B	−141.7 (3)
C7A—Ru1A—O1A—C32A	−70.6 (3)	C5B—Ru1B—O1B—C32B	121.5 (3)
C8A—Ru1A—O1A—C32A	−81.0 (6)	C7B—Ru1B—O1B—C32B	58.8 (3)
C6A—Ru1A—O1A—C32A	−42.3 (3)	C8B—Ru1B—O1B—C32B	30.7 (3)
C4A—Ru1A—O1A—C32A	27.1 (3)	C4B—Ru1B—O1B—C32B	115.3 (3)
C9A—Ru1A—O1A—C32A	49.6 (4)	C6B—Ru1B—O1B—C32B	96.3 (3)
C5A—Ru1A—O1A—C32A	−4.5 (3)	C9B—Ru1B—O1B—C32B	25.0 (4)
O1A—Ru1A—O3A—C39A	−64.8 (3)	O1B—Ru1B—O3B—C39B	108.7 (3)
C1A—Ru1A—O3A—C39A	−143.2 (3)	C1B—Ru1B—O3B—C39B	−161.5 (3)
C7A—Ru1A—O3A—C39A	118.6 (3)	C5B—Ru1B—O3B—C39B	−83.9 (4)
C8A—Ru1A—O3A—C39A	118.3 (3)	C7B—Ru1B—O3B—C39B	27.0 (3)
C6A—Ru1A—O3A—C39A	27.7 (5)	C8B—Ru1B—O3B—C39B	−3.7 (3)
C4A—Ru1A—O3A—C39A	53.6 (3)	C4B—Ru1B—O3B—C39B	−70.5 (3)
C9A—Ru1A—O3A—C39A	91.4 (3)	C6B—Ru1B—O3B—C39B	45.1 (5)
C5A—Ru1A—O3A—C39A	27.8 (3)	C9B—Ru1B—O3B—C39B	−41.5 (3)
C3A—N2A—C1A—N1A	0.5 (4)	C2B—N1B—C1B—N2B	1.2 (4)
C23A—N2A—C1A—N1A	−167.9 (3)	C14B—N1B—C1B—N2B	−167.4 (3)
C3A—N2A—C1A—Ru1A	−167.7 (2)	C2B—N1B—C1B—Ru1B	−167.7 (2)
C23A—N2A—C1A—Ru1A	23.9 (5)	C14B—N1B—C1B—Ru1B	23.8 (5)
C2A—N1A—C1A—N2A	−0.5 (4)	C3B—N2B—C1B—N1B	−0.8 (4)
C14A—N1A—C1A—N2A	170.3 (3)	C23B—N2B—C1B—N1B	169.8 (3)
C2A—N1A—C1A—Ru1A	167.3 (3)	C3B—N2B—C1B—Ru1B	167.7 (2)
C14A—N1A—C1A—Ru1A	−22.0 (5)	C23B—N2B—C1B—Ru1B	−21.7 (5)
O1A—Ru1A—C1A—N2A	80.1 (3)	O3B—Ru1B—C1B—N1B	83.4 (3)
O3A—Ru1A—C1A—N2A	158.8 (3)	O1B—Ru1B—C1B—N1B	161.8 (3)
C7A—Ru1A—C1A—N2A	−49.6 (3)	C5B—Ru1B—C1B—N1B	−78.3 (3)
C8A—Ru1A—C1A—N2A	−87.2 (3)	C7B—Ru1B—C1B—N1B	−117.6 (4)
C6A—Ru1A—C1A—N2A	−16.7 (3)	C8B—Ru1B—C1B—N1B	0.6 (5)
C4A—Ru1A—C1A—N2A	−115.0 (5)	C4B—Ru1B—C1B—N1B	−40.5 (3)
C9A—Ru1A—C1A—N2A	−114.9 (3)	C6B—Ru1B—C1B—N1B	−108.7 (3)
C5A—Ru1A—C1A—N2A	−3.0 (5)	C9B—Ru1B—C1B—N1B	−10.6 (3)
O1A—Ru1A—C1A—N1A	−85.0 (3)	O3B—Ru1B—C1B—N2B	−82.6 (3)
O3A—Ru1A—C1A—N1A	−6.2 (3)	O1B—Ru1B—C1B—N2B	−4.1 (3)
C7A—Ru1A—C1A—N1A	145.3 (3)	C5B—Ru1B—C1B—N2B	115.7 (3)
C8A—Ru1A—C1A—N1A	107.8 (3)	C7B—Ru1B—C1B—N2B	76.5 (5)
C6A—Ru1A—C1A—N1A	178.2 (3)	C8B—Ru1B—C1B—N2B	−165.3 (3)
C4A—Ru1A—C1A—N1A	79.9 (6)	C4B—Ru1B—C1B—N2B	153.6 (3)
C9A—Ru1A—C1A—N1A	80.1 (3)	C6B—Ru1B—C1B—N2B	85.4 (3)
C5A—Ru1A—C1A—N1A	−168.0 (3)	C9B—Ru1B—C1B—N2B	−176.6 (3)
C1A—N1A—C2A—C3A	0.4 (4)	C1B—N1B—C2B—C3B	−1.2 (4)
C14A—N1A—C2A—C3A	−171.8 (3)	C14B—N1B—C2B—C3B	168.6 (3)
N1A—C2A—C3A—N2A	−0.1 (4)	N1B—C2B—C3B—N2B	0.6 (4)
C1A—N2A—C3A—C2A	−0.3 (4)	C1B—N2B—C3B—C2B	0.1 (4)
C23A—N2A—C3A—C2A	169.8 (3)	C23B—N2B—C3B—C2B	−171.7 (3)
O1A—Ru1A—C4A—C5A	−61.6 (2)	O3B—Ru1B—C4B—C9B	53.4 (2)
O3A—Ru1A—C4A—C5A	−139.0 (2)	O1B—Ru1B—C4B—C9B	−124.6 (3)
C1A—Ru1A—C4A—C5A	135.2 (5)	C1B—Ru1B—C4B—C9B	134.3 (2)
C7A—Ru1A—C4A—C5A	67.4 (2)	C5B—Ru1B—C4B—C9B	−133.5 (3)
C8A—Ru1A—C4A—C5A	105.0 (3)	C7B—Ru1B—C4B—C9B	−66.4 (2)
C6A—Ru1A—C4A—C5A	29.1 (2)	C8B—Ru1B—C4B—C9B	−30.0 (2)
C9A—Ru1A—C4A—C5A	135.0 (3)	C6B—Ru1B—C4B—C9B	−104.0 (2)
O1A—Ru1A—C4A—C9A	163.4 (2)	O3B—Ru1B—C4B—C5B	−173.06 (18)
O3A—Ru1A—C4A—C9A	86.1 (2)	O1B—Ru1B—C4B—C5B	8.9 (4)
C1A—Ru1A—C4A—C9A	0.2 (6)	C1B—Ru1B—C4B—C5B	−92.2 (2)
C7A—Ru1A—C4A—C9A	−67.6 (3)	C7B—Ru1B—C4B—C5B	67.2 (2)
C8A—Ru1A—C4A—C9A	−30.0 (3)	C8B—Ru1B—C4B—C5B	103.6 (2)
C6A—Ru1A—C4A—C9A	−105.9 (3)	C6B—Ru1B—C4B—C5B	29.5 (2)
C5A—Ru1A—C4A—C9A	−135.0 (3)	C9B—Ru1B—C4B—C5B	133.5 (3)
C9A—C4A—C5A—C6A	−1.0 (5)	C9B—C4B—C5B—C6B	−2.8 (5)
Ru1A—C4A—C5A—C6A	−53.5 (3)	Ru1B—C4B—C5B—C6B	−56.7 (3)
C9A—C4A—C5A—Ru1A	52.5 (3)	C9B—C4B—C5B—Ru1B	53.9 (3)
O1A—Ru1A—C5A—C4A	128.3 (2)	O3B—Ru1B—C5B—C4B	18.2 (5)
O3A—Ru1A—C5A—C4A	47.4 (2)	O1B—Ru1B—C5B—C4B	−176.03 (18)
C1A—Ru1A—C5A—C4A	−153.1 (3)	C1B—Ru1B—C5B—C4B	92.8 (2)
C7A—Ru1A—C5A—C4A	−101.3 (3)	C7B—Ru1B—C5B—C4B	−101.3 (2)
C8A—Ru1A—C5A—C4A	−64.5 (2)	C8B—Ru1B—C5B—C4B	−65.0 (2)
C6A—Ru1A—C5A—C4A	−132.7 (3)	C6B—Ru1B—C5B—C4B	−131.7 (3)
C9A—Ru1A—C5A—C4A	−28.0 (2)	C9B—Ru1B—C5B—C4B	−27.91 (19)
O1A—Ru1A—C5A—C6A	−98.9 (2)	O3B—Ru1B—C5B—C6B	149.9 (3)
O3A—Ru1A—C5A—C6A	−179.9 (2)	O1B—Ru1B—C5B—C6B	−44.3 (2)
C1A—Ru1A—C5A—C6A	−20.4 (4)	C1B—Ru1B—C5B—C6B	−135.5 (2)
C7A—Ru1A—C5A—C6A	31.4 (3)	C7B—Ru1B—C5B—C6B	30.4 (2)
C8A—Ru1A—C5A—C6A	68.3 (3)	C8B—Ru1B—C5B—C6B	66.7 (2)
C4A—Ru1A—C5A—C6A	132.7 (3)	C4B—Ru1B—C5B—C6B	131.7 (3)
C9A—Ru1A—C5A—C6A	104.7 (3)	C9B—Ru1B—C5B—C6B	103.8 (2)
C4A—C5A—C6A—C7A	−0.9 (5)	C4B—C5B—C6B—C7B	2.4 (5)
Ru1A—C5A—C6A—C7A	−54.8 (3)	Ru1B—C5B—C6B—C7B	−54.3 (3)
C4A—C5A—C6A—C10A	−177.4 (3)	C4B—C5B—C6B—C11B	−175.5 (3)
Ru1A—C5A—C6A—C10A	128.7 (4)	Ru1B—C5B—C6B—C11B	127.8 (3)
C4A—C5A—C6A—Ru1A	53.8 (3)	C4B—C5B—C6B—Ru1B	56.7 (3)
O1A—Ru1A—C6A—C5A	87.4 (2)	O3B—Ru1B—C6B—C5B	−156.0 (3)
O3A—Ru1A—C6A—C5A	0.2 (5)	O1B—Ru1B—C6B—C5B	142.8 (2)
C1A—Ru1A—C6A—C5A	169.9 (2)	C1B—Ru1B—C6B—C5B	55.2 (3)
C7A—Ru1A—C6A—C5A	−128.8 (4)	C7B—Ru1B—C6B—C5B	−130.0 (3)
C8A—Ru1A—C6A—C5A	−101.3 (3)	C8B—Ru1B—C6B—C5B	−102.1 (2)
C4A—Ru1A—C6A—C5A	−28.2 (2)	C4B—Ru1B—C6B—C5B	−29.5 (2)
C9A—Ru1A—C6A—C5A	−65.0 (2)	C9B—Ru1B—C6B—C5B	−65.4 (2)
O1A—Ru1A—C6A—C7A	−143.8 (2)	O3B—Ru1B—C6B—C7B	−26.0 (4)
O3A—Ru1A—C6A—C7A	129.0 (3)	O1B—Ru1B—C6B—C7B	−87.2 (2)
C1A—Ru1A—C6A—C7A	−61.3 (3)	C1B—Ru1B—C6B—C7B	−174.8 (2)
C8A—Ru1A—C6A—C7A	27.6 (2)	C5B—Ru1B—C6B—C7B	130.0 (3)
C4A—Ru1A—C6A—C7A	100.6 (3)	C8B—Ru1B—C6B—C7B	27.9 (2)
C9A—Ru1A—C6A—C7A	63.9 (2)	C4B—Ru1B—C6B—C7B	100.6 (2)
C5A—Ru1A—C6A—C7A	128.8 (4)	C9B—Ru1B—C6B—C7B	64.7 (2)
O1A—Ru1A—C6A—C10A	−26.8 (4)	O3B—Ru1B—C6B—C11B	90.4 (4)
O3A—Ru1A—C6A—C10A	−114.0 (4)	O1B—Ru1B—C6B—C11B	29.2 (3)
C1A—Ru1A—C6A—C10A	55.7 (4)	C1B—Ru1B—C6B—C11B	−58.4 (4)
C7A—Ru1A—C6A—C10A	117.0 (5)	C5B—Ru1B—C6B—C11B	−113.6 (4)
C8A—Ru1A—C6A—C10A	144.5 (4)	C7B—Ru1B—C6B—C11B	116.4 (4)
C4A—Ru1A—C6A—C10A	−142.4 (4)	C8B—Ru1B—C6B—C11B	144.3 (4)
C9A—Ru1A—C6A—C10A	−179.2 (4)	C4B—Ru1B—C6B—C11B	−143.0 (4)
C5A—Ru1A—C6A—C10A	−114.2 (5)	C9B—Ru1B—C6B—C11B	−178.9 (4)
C5A—C6A—C7A—C8A	2.3 (5)	C5B—C6B—C7B—C8B	−0.1 (5)
C10A—C6A—C7A—C8A	178.7 (4)	C11B—C6B—C7B—C8B	177.8 (3)
Ru1A—C6A—C7A—C8A	−53.5 (3)	Ru1B—C6B—C7B—C8B	−53.7 (3)
C5A—C6A—C7A—Ru1A	55.8 (3)	C5B—C6B—C7B—Ru1B	53.6 (3)
C10A—C6A—C7A—Ru1A	−127.8 (4)	C11B—C6B—C7B—Ru1B	−128.5 (3)
O1A—Ru1A—C7A—C8A	−176.19 (19)	O3B—Ru1B—C7B—C8B	−57.9 (2)
O3A—Ru1A—C7A—C8A	−0.5 (4)	O1B—Ru1B—C7B—C8B	−133.5 (2)
C1A—Ru1A—C7A—C8A	−97.0 (2)	C1B—Ru1B—C7B—C8B	146.1 (3)
C6A—Ru1A—C7A—C8A	134.7 (3)	C5B—Ru1B—C7B—C8B	103.2 (2)
C4A—Ru1A—C7A—C8A	67.5 (2)	C4B—Ru1B—C7B—C8B	65.6 (2)
C9A—Ru1A—C7A—C8A	29.3 (2)	C6B—Ru1B—C7B—C8B	133.7 (3)
C5A—Ru1A—C7A—C8A	103.8 (2)	C9B—Ru1B—C7B—C8B	29.2 (2)
O1A—Ru1A—C7A—C6A	49.1 (3)	O3B—Ru1B—C7B—C6B	168.38 (19)
O3A—Ru1A—C7A—C6A	−135.2 (3)	O1B—Ru1B—C7B—C6B	92.8 (2)
C1A—Ru1A—C7A—C6A	128.3 (2)	C1B—Ru1B—C7B—C6B	12.4 (5)
C8A—Ru1A—C7A—C6A	−134.7 (3)	C5B—Ru1B—C7B—C6B	−30.5 (2)
C4A—Ru1A—C7A—C6A	−67.3 (2)	C8B—Ru1B—C7B—C6B	−133.7 (3)
C9A—Ru1A—C7A—C6A	−105.4 (2)	C4B—Ru1B—C7B—C6B	−68.1 (2)
C5A—Ru1A—C7A—C6A	−31.0 (2)	C9B—Ru1B—C7B—C6B	−104.5 (2)
C6A—C7A—C8A—C9A	−1.8 (6)	C6B—C7B—C8B—C9B	−1.9 (5)
Ru1A—C7A—C8A—C9A	−55.5 (3)	Ru1B—C7B—C8B—C9B	−55.6 (3)
C6A—C7A—C8A—Ru1A	53.7 (3)	C6B—C7B—C8B—Ru1B	53.7 (3)
O1A—Ru1A—C8A—C9A	144.6 (5)	O3B—Ru1B—C8B—C7B	132.7 (2)
O3A—Ru1A—C8A—C9A	−48.8 (3)	O1B—Ru1B—C8B—C7B	52.2 (2)
C1A—Ru1A—C8A—C9A	−140.8 (3)	C1B—Ru1B—C8B—C7B	−148.4 (3)
C7A—Ru1A—C8A—C9A	131.5 (3)	C5B—Ru1B—C8B—C7B	−65.9 (2)
C6A—Ru1A—C8A—C9A	103.5 (3)	C4B—Ru1B—C8B—C7B	−103.0 (2)
C4A—Ru1A—C8A—C9A	30.7 (2)	C6B—Ru1B—C8B—C7B	−28.5 (2)
C5A—Ru1A—C8A—C9A	66.2 (3)	C9B—Ru1B—C8B—C7B	−132.4 (3)
O1A—Ru1A—C8A—C7A	13.1 (6)	O3B—Ru1B—C8B—C9B	−94.9 (2)
O3A—Ru1A—C8A—C7A	179.76 (19)	O1B—Ru1B—C8B—C9B	−175.43 (19)
C1A—Ru1A—C8A—C7A	87.7 (2)	C1B—Ru1B—C8B—C9B	−16.0 (4)
C6A—Ru1A—C8A—C7A	−28.0 (2)	C5B—Ru1B—C8B—C9B	66.4 (2)
C4A—Ru1A—C8A—C7A	−100.7 (2)	C7B—Ru1B—C8B—C9B	132.4 (3)
C9A—Ru1A—C8A—C7A	−131.5 (3)	C4B—Ru1B—C8B—C9B	29.4 (2)
C5A—Ru1A—C8A—C7A	−65.3 (2)	C6B—Ru1B—C8B—C9B	103.9 (2)
C7A—C8A—C9A—C4A	−0.2 (5)	C5B—C4B—C9B—C8B	0.7 (5)
Ru1A—C8A—C9A—C4A	−54.8 (3)	Ru1B—C4B—C9B—C8B	53.1 (3)
C7A—C8A—C9A—C11A	−177.8 (4)	C5B—C4B—C9B—C10B	177.5 (3)
Ru1A—C8A—C9A—C11A	127.6 (4)	Ru1B—C4B—C9B—C10B	−130.1 (3)
C7A—C8A—C9A—Ru1A	54.6 (3)	C5B—C4B—C9B—Ru1B	−52.4 (3)
C5A—C4A—C9A—C8A	1.6 (5)	C7B—C8B—C9B—C4B	1.6 (5)
Ru1A—C4A—C9A—C8A	54.6 (3)	Ru1B—C8B—C9B—C4B	−53.4 (3)
C5A—C4A—C9A—C11A	179.3 (3)	C7B—C8B—C9B—C10B	−175.2 (3)
Ru1A—C4A—C9A—C11A	−127.7 (3)	Ru1B—C8B—C9B—C10B	129.7 (3)
C5A—C4A—C9A—Ru1A	−53.0 (3)	C7B—C8B—C9B—Ru1B	55.1 (3)
O1A—Ru1A—C9A—C8A	−163.5 (3)	O3B—Ru1B—C9B—C4B	−137.9 (2)
O3A—Ru1A—C9A—C8A	136.6 (3)	O1B—Ru1B—C9B—C4B	139.6 (2)
C1A—Ru1A—C9A—C8A	50.0 (3)	C1B—Ru1B—C9B—C4B	−55.9 (2)
C7A—Ru1A—C9A—C8A	−29.7 (3)	C5B—Ru1B—C9B—C4B	28.6 (2)
C6A—Ru1A—C9A—C8A	−66.7 (3)	C7B—Ru1B—C9B—C4B	102.4 (2)
C4A—Ru1A—C9A—C8A	−130.1 (4)	C8B—Ru1B—C9B—C4B	131.0 (3)
C5A—Ru1A—C9A—C8A	−103.2 (3)	C6B—Ru1B—C9B—C4B	65.6 (2)
O1A—Ru1A—C9A—C4A	−33.4 (4)	O3B—Ru1B—C9B—C8B	91.1 (2)
O3A—Ru1A—C9A—C4A	−93.3 (2)	O1B—Ru1B—C9B—C8B	8.6 (4)
C1A—Ru1A—C9A—C4A	−179.9 (2)	C1B—Ru1B—C9B—C8B	173.03 (19)
C7A—Ru1A—C9A—C4A	100.4 (3)	C5B—Ru1B—C9B—C8B	−102.4 (2)
C8A—Ru1A—C9A—C4A	130.1 (4)	C7B—Ru1B—C9B—C8B	−28.7 (2)
C6A—Ru1A—C9A—C4A	63.4 (2)	C4B—Ru1B—C9B—C8B	−131.0 (3)
C5A—Ru1A—C9A—C4A	26.9 (2)	C6B—Ru1B—C9B—C8B	−65.4 (2)
O1A—Ru1A—C9A—C11A	76.8 (5)	O3B—Ru1B—C9B—C10B	−22.7 (4)
O3A—Ru1A—C9A—C11A	16.9 (4)	O1B—Ru1B—C9B—C10B	−105.2 (4)
C1A—Ru1A—C9A—C11A	−69.7 (4)	C1B—Ru1B—C9B—C10B	59.3 (4)
C7A—Ru1A—C9A—C11A	−149.3 (4)	C5B—Ru1B—C9B—C10B	143.8 (4)
C8A—Ru1A—C9A—C11A	−119.7 (5)	C7B—Ru1B—C9B—C10B	−142.4 (4)
C6A—Ru1A—C9A—C11A	173.7 (4)	C8B—Ru1B—C9B—C10B	−113.8 (4)
C4A—Ru1A—C9A—C11A	110.3 (5)	C4B—Ru1B—C9B—C10B	115.2 (4)
C5A—Ru1A—C9A—C11A	137.1 (4)	C6B—Ru1B—C9B—C10B	−179.2 (4)
C8A—C9A—C11A—C13A	−27.9 (6)	C5B—C6B—C11B—C12B	174.2 (4)
C4A—C9A—C11A—C13A	154.5 (4)	C7B—C6B—C11B—C12B	−3.6 (5)
Ru1A—C9A—C11A—C13A	66.2 (5)	Ru1B—C6B—C11B—C12B	−96.2 (4)
C8A—C9A—C11A—C12A	97.4 (5)	C5B—C6B—C11B—C13B	54.3 (5)
C4A—C9A—C11A—C12A	−80.2 (5)	C7B—C6B—C11B—C13B	−123.6 (4)
Ru1A—C9A—C11A—C12A	−168.5 (3)	Ru1B—C6B—C11B—C13B	143.8 (4)
C1A—N1A—C14A—C15A	−81.6 (5)	C1B—N1B—C14B—C19B	−99.3 (4)
C2A—N1A—C14A—C15A	88.8 (4)	C2B—N1B—C14B—C19B	92.9 (4)
C1A—N1A—C14A—C19A	109.5 (4)	C1B—N1B—C14B—C15B	87.6 (4)
C2A—N1A—C14A—C19A	−80.1 (4)	C2B—N1B—C14B—C15B	−80.3 (4)
C19A—C14A—C15A—C16A	−4.0 (6)	C19B—C14B—C15B—C16B	3.6 (5)
N1A—C14A—C15A—C16A	−172.5 (4)	N1B—C14B—C15B—C16B	176.6 (3)
C19A—C14A—C15A—C20A	173.2 (4)	C19B—C14B—C15B—C20B	−178.0 (3)
N1A—C14A—C15A—C20A	4.7 (6)	N1B—C14B—C15B—C20B	−4.9 (5)
C14A—C15A—C16A—C17A	1.0 (7)	C14B—C15B—C16B—C17B	−0.7 (5)
C20A—C15A—C16A—C17A	−176.2 (5)	C20B—C15B—C16B—C17B	−179.2 (3)
C15A—C16A—C17A—C18A	0.9 (7)	C15B—C16B—C17B—C18B	−2.2 (5)
C15A—C16A—C17A—C21A	−179.9 (5)	C15B—C16B—C17B—C21B	179.0 (3)
C16A—C17A—C18A—C19A	0.1 (6)	C16B—C17B—C18B—C19B	2.4 (6)
C21A—C17A—C18A—C19A	−179.1 (4)	C21B—C17B—C18B—C19B	−178.8 (4)
C17A—C18A—C19A—C14A	−2.9 (5)	C17B—C18B—C19B—C14B	0.3 (5)
C17A—C18A—C19A—C22A	176.9 (4)	C17B—C18B—C19B—C22B	−179.4 (3)
C15A—C14A—C19A—C18A	4.9 (5)	C15B—C14B—C19B—C18B	−3.4 (5)
N1A—C14A—C19A—C18A	173.4 (3)	N1B—C14B—C19B—C18B	−176.3 (3)
C15A—C14A—C19A—C22A	−174.9 (4)	C15B—C14B—C19B—C22B	176.3 (3)
N1A—C14A—C19A—C22A	−6.4 (5)	N1B—C14B—C19B—C22B	3.4 (5)
C1A—N2A—C23A—C24A	−104.4 (4)	C1B—N2B—C23B—C24B	−71.3 (4)
C3A—N2A—C23A—C24A	87.7 (4)	C3B—N2B—C23B—C24B	98.7 (4)
C1A—N2A—C23A—C28A	85.5 (4)	C1B—N2B—C23B—C28B	114.9 (4)
C3A—N2A—C23A—C28A	−82.4 (4)	C3B—N2B—C23B—C28B	−75.0 (4)
C28A—C23A—C24A—C25A	−2.8 (5)	C28B—C23B—C24B—C25B	−2.1 (5)
N2A—C23A—C24A—C25A	−172.5 (3)	N2B—C23B—C24B—C25B	−175.6 (3)
C28A—C23A—C24A—C31A	175.5 (3)	C28B—C23B—C24B—C29B	175.9 (3)
N2A—C23A—C24A—C31A	5.8 (5)	N2B—C23B—C24B—C29B	2.4 (5)
C23A—C24A—C25A—C26A	0.4 (5)	C23B—C24B—C25B—C26B	1.8 (5)
C31A—C24A—C25A—C26A	−178.0 (3)	C29B—C24B—C25B—C26B	−176.2 (4)
C24A—C25A—C26A—C27A	1.3 (5)	C24B—C25B—C26B—C27B	0.3 (5)
C24A—C25A—C26A—C30A	−179.3 (3)	C24B—C25B—C26B—C30B	178.5 (4)
C25A—C26A—C27A—C28A	−0.7 (5)	C25B—C26B—C27B—C28B	−2.2 (5)
C30A—C26A—C27A—C28A	179.9 (3)	C30B—C26B—C27B—C28B	179.6 (3)
C26A—C27A—C28A—C23A	−1.6 (5)	C26B—C27B—C28B—C23B	1.9 (5)
C26A—C27A—C28A—C29A	178.8 (3)	C26B—C27B—C28B—C31B	−176.5 (3)
C24A—C23A—C28A—C27A	3.4 (5)	C24B—C23B—C28B—C27B	0.3 (5)
N2A—C23A—C28A—C27A	173.3 (3)	N2B—C23B—C28B—C27B	173.8 (3)
C24A—C23A—C28A—C29A	−176.9 (3)	C24B—C23B—C28B—C31B	178.7 (3)
N2A—C23A—C28A—C29A	−7.1 (5)	N2B—C23B—C28B—C31B	−7.8 (5)
Ru1A—O1A—C32A—O2A	16.4 (5)	Ru1B—O1B—C32B—O2B	−6.0 (5)
Ru1A—O1A—C32A—C33A	−164.0 (2)	Ru1B—O1B—C32B—C33B	174.4 (2)
O2A—C32A—C33A—C38A	−27.3 (5)	O2B—C32B—C33B—C34B	63.6 (5)
O1A—C32A—C33A—C38A	153.1 (3)	O1B—C32B—C33B—C34B	−116.8 (4)
O2A—C32A—C33A—C34A	153.7 (3)	O2B—C32B—C33B—C38B	−112.8 (4)
O1A—C32A—C33A—C34A	−25.9 (5)	O1B—C32B—C33B—C38B	66.9 (4)
C38A—C33A—C34A—F1A	175.1 (3)	C38B—C33B—C34B—F1B	179.0 (3)
C32A—C33A—C34A—F1A	−5.8 (5)	C32B—C33B—C34B—F1B	2.5 (5)
C38A—C33A—C34A—C35A	−2.6 (5)	C38B—C33B—C34B—C35B	−0.7 (5)
C32A—C33A—C34A—C35A	176.5 (3)	C32B—C33B—C34B—C35B	−177.2 (3)
F1A—C34A—C35A—F2A	2.9 (5)	F1B—C34B—C35B—F2B	0.8 (5)
C33A—C34A—C35A—F2A	−179.3 (3)	C33B—C34B—C35B—F2B	−179.5 (3)
F1A—C34A—C35A—C36A	−175.5 (3)	F1B—C34B—C35B—C36B	−178.9 (3)
C33A—C34A—C35A—C36A	2.4 (6)	C33B—C34B—C35B—C36B	0.8 (5)
F2A—C35A—C36A—F3A	0.3 (5)	F2B—C35B—C36B—F3B	0.6 (5)
C34A—C35A—C36A—F3A	178.7 (3)	C34B—C35B—C36B—F3B	−179.7 (3)
F2A—C35A—C36A—C37A	−179.3 (3)	F2B—C35B—C36B—C37B	−179.0 (4)
C34A—C35A—C36A—C37A	−0.9 (5)	C34B—C35B—C36B—C37B	0.7 (6)
F3A—C36A—C37A—F4A	−0.6 (5)	F3B—C36B—C37B—F4B	−1.9 (6)
C35A—C36A—C37A—F4A	179.0 (3)	C35B—C36B—C37B—F4B	177.8 (4)
F3A—C36A—C37A—C38A	−179.7 (3)	F3B—C36B—C37B—C38B	178.2 (3)
C35A—C36A—C37A—C38A	−0.1 (5)	C35B—C36B—C37B—C38B	−2.2 (6)
F4A—C37A—C38A—F5A	0.9 (5)	F4B—C37B—C38B—F5B	1.3 (6)
C36A—C37A—C38A—F5A	−180.0 (3)	C36B—C37B—C38B—F5B	−178.7 (3)
F4A—C37A—C38A—C33A	−179.3 (3)	F4B—C37B—C38B—C33B	−177.7 (3)
C36A—C37A—C38A—C33A	−0.2 (6)	C36B—C37B—C38B—C33B	2.3 (6)
C34A—C33A—C38A—F5A	−178.7 (3)	C34B—C33B—C38B—F5B	−179.8 (3)
C32A—C33A—C38A—F5A	2.2 (5)	C32B—C33B—C38B—F5B	−3.3 (5)
C34A—C33A—C38A—C37A	1.5 (5)	C34B—C33B—C38B—C37B	−0.8 (5)
C32A—C33A—C38A—C37A	−177.6 (3)	C32B—C33B—C38B—C37B	175.7 (3)
Ru1A—O3A—C39A—O4A	−2.9 (6)	Ru1B—O3B—C39B—O4B	17.8 (5)
Ru1A—O3A—C39A—C40A	177.9 (2)	Ru1B—O3B—C39B—C40B	−163.2 (2)
O4A—C39A—C40A—C41A	63.5 (5)	O4B—C39B—C40B—C45B	−32.1 (5)
O3A—C39A—C40A—C41A	−117.3 (4)	O3B—C39B—C40B—C45B	148.8 (3)
O4A—C39A—C40A—C45A	−114.5 (4)	O4B—C39B—C40B—C41B	146.9 (3)
O3A—C39A—C40A—C45A	64.8 (5)	O3B—C39B—C40B—C41B	−32.2 (5)
C45A—C40A—C41A—F6A	179.5 (3)	C45B—C40B—C41B—F6B	178.0 (3)
C39A—C40A—C41A—F6A	1.4 (5)	C39B—C40B—C41B—F6B	−1.1 (5)
C45A—C40A—C41A—C42A	−1.1 (6)	C45B—C40B—C41B—C42B	−1.0 (5)
C39A—C40A—C41A—C42A	−179.2 (3)	C39B—C40B—C41B—C42B	180.0 (3)
F6A—C41A—C42A—F7A	0.5 (5)	F6B—C41B—C42B—F7B	1.6 (5)
C40A—C41A—C42A—F7A	−178.9 (3)	C40B—C41B—C42B—F7B	−179.4 (3)
F6A—C41A—C42A—C43A	−179.2 (3)	F6B—C41B—C42B—C43B	−177.3 (3)
C40A—C41A—C42A—C43A	1.4 (6)	C40B—C41B—C42B—C43B	1.7 (5)
F7A—C42A—C43A—F8A	0.2 (6)	F7B—C42B—C43B—F8B	0.1 (5)
C41A—C42A—C43A—F8A	180.0 (3)	C41B—C42B—C43B—F8B	179.0 (3)
F7A—C42A—C43A—C44A	−179.2 (4)	F7B—C42B—C43B—C44B	−179.6 (3)
C41A—C42A—C43A—C44A	0.6 (6)	C41B—C42B—C43B—C44B	−0.6 (5)
F8A—C43A—C44A—F9A	−0.3 (6)	F8B—C43B—C44B—F9B	−1.0 (6)
C42A—C43A—C44A—F9A	179.1 (4)	C42B—C43B—C44B—F9B	178.6 (3)
F8A—C43A—C44A—C45A	178.0 (4)	F8B—C43B—C44B—C45B	179.3 (3)
C42A—C43A—C44A—C45A	−2.6 (6)	C42B—C43B—C44B—C45B	−1.0 (6)
C41A—C40A—C45A—F10A	−179.1 (3)	F9B—C44B—C45B—F10B	2.0 (5)
C39A—C40A—C45A—F10A	−1.1 (6)	C43B—C44B—C45B—F10B	−178.4 (3)
C41A—C40A—C45A—C44A	−1.0 (6)	F9B—C44B—C45B—C40B	−177.9 (3)
C39A—C40A—C45A—C44A	177.0 (3)	C43B—C44B—C45B—C40B	1.7 (6)
F9A—C44A—C45A—F10A	−0.6 (6)	C41B—C40B—C45B—F10B	179.4 (3)
C43A—C44A—C45A—F10A	−178.9 (4)	C39B—C40B—C45B—F10B	−1.6 (5)
F9A—C44A—C45A—C40A	−178.8 (4)	C41B—C40B—C45B—C44B	−0.8 (5)
C43A—C44A—C45A—C40A	2.9 (6)	C39B—C40B—C45B—C44B	178.3 (3)
